# Efficacy, Immunogenicity, and Safety of COVID-19 Vaccines in Randomized Control Trials in the Pre-Delta Era: A Systematic Review and Network Meta-Analysis

**DOI:** 10.3390/vaccines10101572

**Published:** 2022-09-20

**Authors:** SuA Oh, Sujata Purja, Hocheol Shin, Min Seo Kim, Seoyeon Park, Andreas Kronbichler, Lee Smith, Michael Eisenhut, Jae Il Shin, Eunyoung Kim

**Affiliations:** 1 Data Science, Evidence-Based and Clinical Research Laboratory, Department of Health, Social and Clinical Pharmacy, College of Pharmacy, Chung-Ang University, Seoul 06974, Korea; 2 Genomics and Digital Health, Samsung Advanced Institute for Health Sciences and Technology, Sungkyunkwan University, Seoul 03063, Korea; 3 Yonsei University College of Medicine, Seoul 03722, Korea; 4 Department of Medicine, University of Cambridge, Cambridge CB2 2QQ, UK; 5 Center for Health Performance and Wellbeing, Anglia Ruskin University, Cambridge CB2 1TN, UK; 6 Luton & Dunstable University Hospital, NHS Foundation Trust, Luton LU40DZ, UK; 7 Department of Pediatrics, Yonsei University College of Medicine, Seoul 03722, Korea

**Keywords:** COVID-19, SARS-CoV-2, vaccine, network meta-analysis

## Abstract

The most effective method of limiting the coronavirus disease pandemic of 2019 (COVID-19) is vaccination. For the determination of the comparative efficacy and safety of COVID-19 vaccines and their platforms during the pre-Delta era, a systematic review and network meta-analysis was conducted. The MEDLINE, Embase, and MedRxiv databases were searched, and the gray literature was manually searched up to 8 July 2021. The review includes the phase II and III randomized controlled trials (RCTs) that assessed the efficacy, immunogenicity, and safety of the COVID-19 vaccines. The network meta-analysis used a Bayesian model and used the surface under the cumulative ranking to rank the comparisons between the vaccines. All included studies were quality appraised according to their design, and the heterogeneity of the analyses was assessed using I2. In terms of vaccine efficacy, the mRNA-1273 vaccine ranked the highest, and the CoronaVac vaccine ranked the lowest. The mRNA-1273 ranked the highest for neutralizing antibody responses to live SARS-CoV-2. The WIV04 vaccine was associated with the lowest incidence of both local and systemic adverse reactions. All studies except one had a low to moderate risk of bias. The mRNA platform vaccines showed higher efficacy and more adverse reactions than the other vaccines.

## 1. Introduction

In March 2020, the World Health Organization (WHO) declared the coronavirus disease (COVID-19) 2019 outbreak a pandemic. COVID-19 presents a variety of symptoms, including fever, cough, and anosmia [1,2,3].

RNA viruses have a high mutation rate, and mutations that give the population a disadvantage are likely to spread [4]. SARS-CoV-2 has a base mutation rate of 4 × 10^−4^ nucleotide substitutions per site per year, or roughly one–two mutations each month [5,6]. The WHO has identified the COVID-19 variants of concern. The first variants were alpha and beta, which were prominent at the time of vaccine development [7]. In 2021, the delta variant caused rapid increases in cases and hospitalizations after it was first reported in the Indian state of Maharashtra in late 2020 [8]. Rapid spread is a unique feature of delta, which dominated the majority of the world in 2021, and has been linked to significant outbreaks, even in areas with comparatively good vaccination coverage [9]. Nevertheless, the developed vaccines provided protection against the delta variant after being released [10]. However, the clinical trials of the vaccines developed in the pre-delta era were conducted in situations where the majority of patients were not vaccinated and did not expect an effect on herd immunity. Therefore, studies on the efficacy of the vaccines in the pre-delta era are important for the post-delta era. Studies conducted during this time are significant because they can offer a comparable prediction model for determining or forecasting the efficacy of a vaccine that was initially developed, even if a new epidemic emerges in the future. Additionally, no head-to-head experiments were conducted to compare the efficacy of the initially designed COVID-19 vaccinations. Therefore, it is challenging to directly and fairly assess the efficacy and safety of different vaccines and vaccine delivery systems. Through the use of network meta-analysis (NMA), a comparative analysis of the relative safety and efficacy of vaccines is feasible.

The goal of this systematic review and network meta-analysis was to determine the COVID-19 vaccination efficacy, immunogenicity, and safety in preventing COVID-19 and its spread in the pre-delta era.

## 2. Methods

### 2.1. Search Strategy and Selection Criteria

For this systematic review and network meta-analysis, the MEDLINE, Embase, and MedRxiv databases were searched from the date of their inception to 8 July 2021. Keywords (supplementary p. 4, [11,12,13,14,15,16,17,18,19,20]) were searched in the title, abstract, and under the medical subject headings. The MedRxiv database was also manually searched for in the gray literature.

Only randomized controlled trials (RCTs) that evaluated the efficacy or immunogenicity of the COVID-19 vaccines in participants (age ≥ 16 years) were included. There are currently 22 approved vaccines; however, only 13 have been the subject of published RCT articles. This study, therefore, assessed the published efficacy of 13 vaccines, of which 4 have been approved by the U.S. Food and Drug Administration (FDA) or the European Medicines Agency (EMA), 11 have been licensed in more than one country, and 2 have not yet been approved (Table 1). The names of the vaccines are defined as the generic name; Table S2 also lists the other names (trade name and manufacturer).

The intervention was defined as any COVID-19 vaccine, while the comparator was a placebo only. The language of the publications was limited to English, and those publications without efficacy or immunogenicity data were excluded. A number of the included individual studies employed results from divided groups. This study, thus, reported whether the studies used divided group data (Table 2).

**Figure 1:**
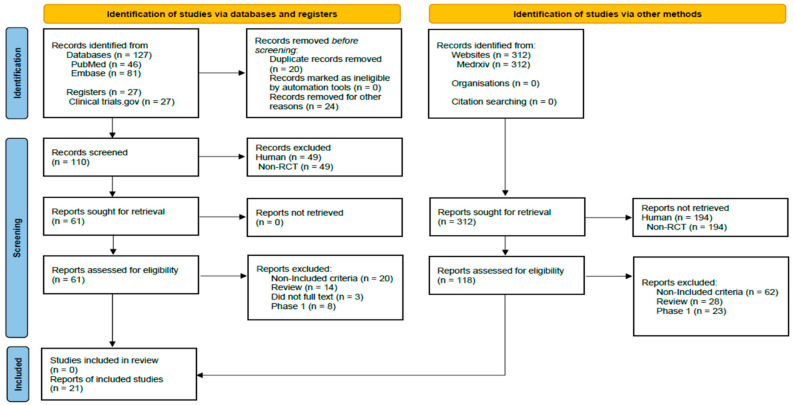
PRISMA flow chart of the article search and screening process.

This study was prepared according to the PRISMA extension statement for network meta-analyses guidelines [21] (Table S1 and Figure 1). The protocol for this systematic review was registered with the International Prospective Register of Systematic Reviews (CRD42021266372).

### 2.2. Outcomes and Data Analysis

The primary outcomes were vaccine efficacy, the immunogenic endpoint of neutralizing antibodies in live SARS-CoV-2, and local and systemic adverse reactions (ARs) to the full-dose regimen. Vaccine efficacy was defined as the vaccine’s efficacy against confirmed COVID-19, with the onset occurring after the last dose in participants who had no serologic or virologic evidence of SARS-CoV-2 infection at the baseline. Confirmed COVID-19 was defined according to the FDA criteria as symptoms of cough and fever [3]. Most studies measured immunogenicity 14 days after the last dose; however, this study included those studies that measured immunogenicity on the day of the last dose or more than 14 days after the last dose. Table S4 provides more information. The secondary outcomes were the geometric mean titers of neutralizing antibodies or specific immunoglobulin G, the unsolicited local and systemic ARs of the first and second vaccinations, and serious adverse events (SAEs). The definition of “safety” is given in Table S3.

This study performed a network meta-analysis of indirect treatment comparisons between the vaccines. Indirect treatment comparisons were performed using the Bayesian models with the established method outlined by the National Institute for Health and Care Excellence [11]. Network meta-analyses provide more precise estimates than standard pairwise analyses [22] and can rank treatments to inform clinical decisions [23]. This study also used the frequentist model to check the correctness of the Bayesian model. The analysis was conducted using the statistical package in R studio (version 4.1.1).

**Figure 2:**
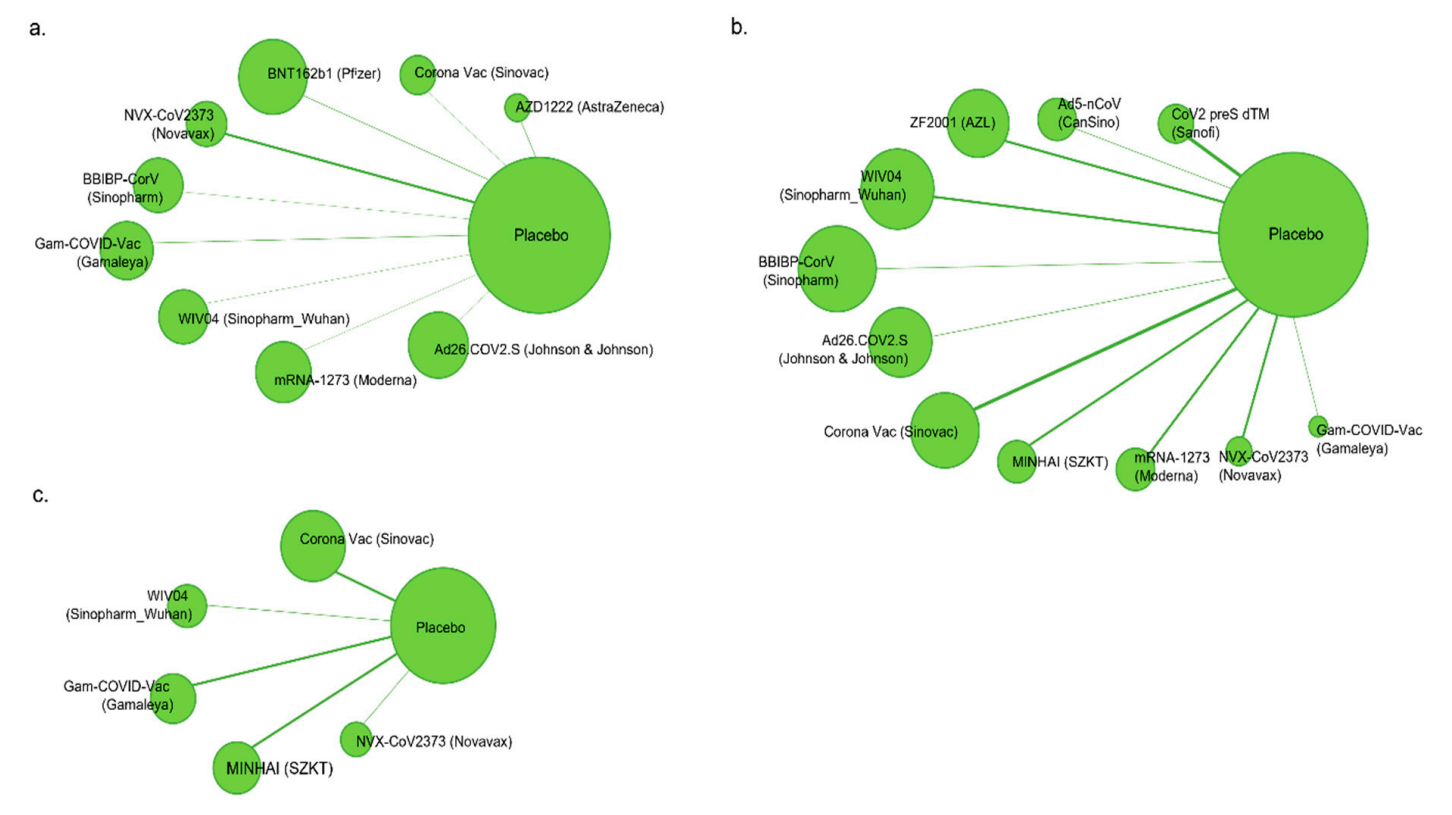
Network plots for indirect comparison of vaccine efficacy and immunogenicity. The size of the nodes is proportional to the number of subjects (sample size) randomly chosen to receive the therapy. The width of the lines is proportional to the number of trials comparing each pair of treatments. (**a**) Vaccine efficacy; (**b**) neutralizing antibodies to live SARS-CoV-2; (**c**) neutralizing antibody to specific and immunoglobulin G.

The symmetry and geometry of the pieces of evidence were examined using a network plot with a node size corresponding to the number of study participants and a connection size corresponding to the number of studies (Figure 2 and Figure S1).

The quality assessment, which included individual articles, was performed using the Cochrane risk of bias tool RoB2. In addition, the quality of the evidence of collective outcomes was estimated using the grading of recommendations assessment, development, and evaluation (GRADE) framework [24]. Publication bias was also assessed using funnel plots [25]. Vaccine efficacy is expressed as the pooled relative risk (RR) and 95% confidence interval (CI). Immunogenicity is expressed as the standard mean difference (SMD) and 95% CI, while the safety outcomes are expressed as RR and 95% CI. All analyses used a random-effects model as a conservative estimate (Table S24). Due to large deviation errors, only the frequentist models were used to analyze IgG antibody responses.

This study assessed the statistical heterogeneity across all comparisons using the I^2^ measure from the netmeta and GeMTC package. The I^2^ value ranged from 0–100%. The heterogeneity levels are low for values of 25–49%, moderate for those of 50–74%, and high for those >75% [26]. This study performed a meta-regression to identify the causes and trends for high heterogeneity based on the baseline characteristics. It also conducted a subgroup analysis to compare the vaccine platforms. The interventions were ranked according to their P-score and surface under the cumulative ranking (SUCRA), which ranges from 0–1 [23]. The P-score and SUCRAs are based solely on the point estimates and standard errors of the network estimates and measure the mean extent of the network estimates and the mean extent of certainty that one intervention is superior to another after being averaged over all competing interventions. When interpreting the results, however, it is also important to take the RR, SMD, and corresponding 95% CI for each comparison into account rather than solely relying on rankings [27]. The Supplementary Material provides more detailed information (p. 4).

## 3. Results

**Figure 3:**
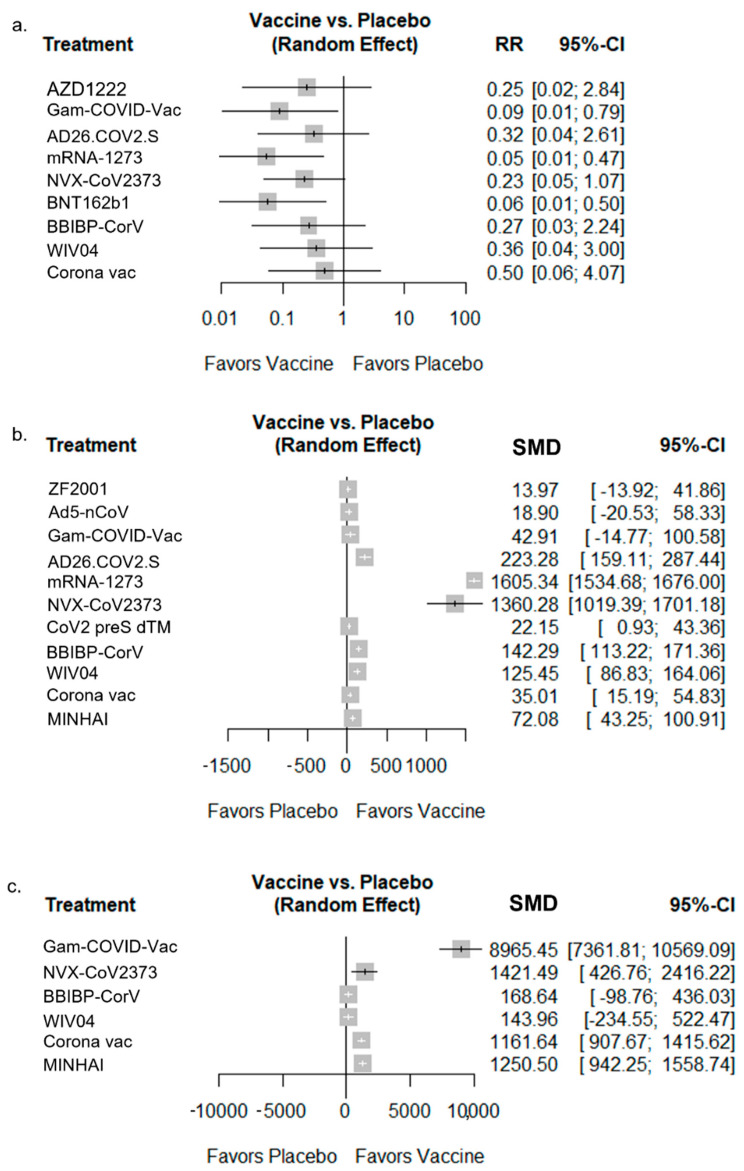
Forest plots for efficacy and immunogenicity of diverse COVID-19 vaccines compared to a placebo. (**a**) Vaccine efficacy; (**b**) neutralizing antibodies to live SARS-CoV-2; (**c**) neutralizing antibody to specific and immunoglobulin G.

Vaccine efficacy was assessed on 206,434 participants in nine RCTs. A total of three of the vaccines (mRNA-1273, BNT162b2, and Gam-COVID-Vac) were significantly more effective than the placebo (Figure 3a and Table S5); the other vaccines showed higher efficacy than the placebo, but the differences were not statistically significant. The heterogeneity of vaccine efficacy is shown in Table S7, with the CoronaVac (Sinovac) vaccines showing the lowest relative efficacy. More detailed results are given in Table S25.

This study included 14 RCTs that assessed the neutralizing antibody response to live SARS-CoV-2, covering 11 vaccines and a total of 10,208 participants. The vaccines had higher levels of neutralizing antibodies to live SARS-CoV-2 than the placebo. The levels of neutralizing antibodies to live SARS-CoV-2 highly increased after the mRNA-1273 and NVX-CoV2373 vaccines. However, some vaccines showed no difference compared with the placebo (Figure 3b and Tables S5 and S26).

For the analysis of specific and IgG antibody responses, 3189 participants were included in six RCTs covering six vaccines (Figure 3c). Gam-COVID-Vac showed the highest statistically significant change in antibody responses. CoronaVac, NVX-CoV2373, and MINHAI also showed statistically significant changes; however, WIV04 showed no statistically significant difference compared with the placebo. As shown in Table S7, immunogenicity corresponded with high heterogeneity (Table S7). More detailed results on immunogenicity are shown in Figure 3 and Table S5.

**Figure 4:**
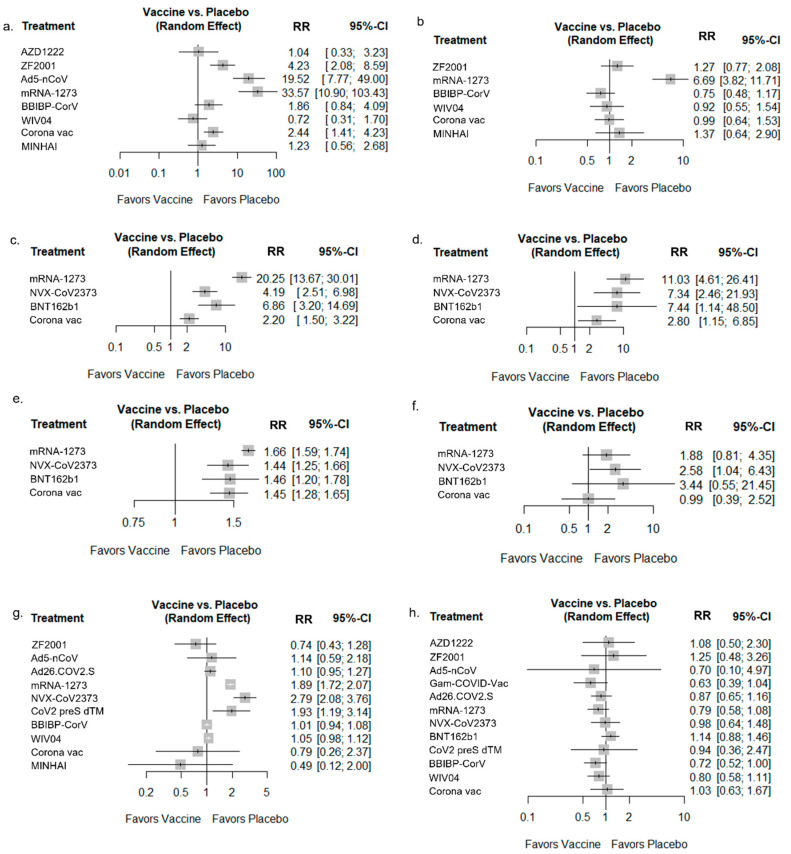
Forest plots for local and systemic ARs, unsolicited ARs, and SAEs compared to the placebo. (**a**) Any local AR to total vaccination; (**b**) any systemic AR to total vaccination; (**c**) any local AR to first vaccination; (**d**) any local AR to second vaccination; (**e**) any systemic AR to first vaccination; (**f**) any systemic AR to second vaccination; (**g**) unsolicited AR; (**h**) SAE.

A total of 11 RCTs from 7 vaccines with a total of 89,444 participants were included in the total dose to local AR analysis (Figure 4a); most of the vaccines were associated with a higher risk of local ARs than the placebo. Eight RCTs from six vaccines with a total of 86,244 participants were included in the analysis of systemic ARs (Figure 4b). A total of three vaccines had fewer systemic ARs than the placebo, although the difference was not statistically significant: BBIBP-CorV (RR: 0.75; 95% CI: 0.48–1/17), WIV04 (RR: 0.92; 95% CI: 0.55–1.54), and CoronaVac (RR: 0.99; 95% CI: 0.64–1.53). However, the other vaccines had more systemic ARs than the placebo. The mRNA-1273 (RR: 6.69; 95% CI: 3.82–11.71) vaccine showed the most ARs among the six vaccines (Figure 4b). The safety results had low to moderate heterogenicity across the studies (Table S7). The detailed safety results are reported in Tables S6 and S27–S34.

**Figure 5:**
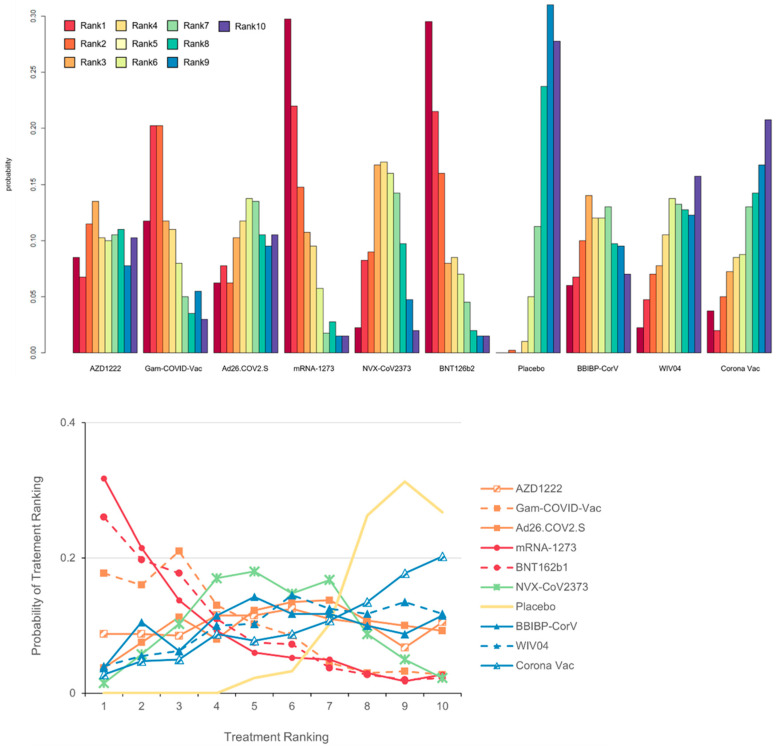
Cumulative ranking of probability for the detection of vaccine efficacy. Changes in the ranking of treatments across different vaccine efficacy scores. Cumulative rank probabilities for each treatment were estimated using SUCRA. Vaccine efficacy is best almost surely when the SUCRA index is 1 and the worst when it equals 0.

Figure 5 shows the cumulative ranking of probability for detecting vaccine efficacy. The mRNA-1273 vaccine had the highest efficacy (SUCRA: 0.77), BNT162b2 had the second highest, and CoronaVac had the lowest efficacy (SUCRA: 0.32). mRNA-1273 and NVXCoV2373 produced the highest neutralizing antibody responses to live SARS-CoV-2 (SUCRAs: 0.99 and 0.92, respectively), and Gam-COVID-Vac and NVX-CoV2373 produced the highest neutralizing antibody responses to specific and IgG responses (P-scores: 1.00 vs. 0.72). Safety SUCRAs and P-scores were similar. Detailed efficacy and safety results are reported in Tables S19–S21.

A subgroup analysis was performed according to the vaccine platform. In terms of efficacy, the mRNA vaccines ranked highest, whereas the inactivated vaccines ranked lowest. The adenovirus-based platform was ranked highest for IgG antibody response, and the mRNA vaccines ranked highest for neutralizing antibody response to live SARS-CoV-2. The inactivated vaccines ranked highest in terms of safety for local and systemic ARs (Figures S2 and S3, Table S19). This study found covariates that could explain the high heterogeneity in most of the meta-regression (Figures S7 and S8 and Tables S22 and S23).

The overall risk of bias was low to moderate, except for one study. Figure S6 illustrates the quality of evidence using ROB2 (Figures S4 and S5), and the certainty of evidence (GRADE) for each outcome is summarized in Table 3 and Table S35. The results of the funnel plot to assess publication bias are shown in Tables S4 and S5. The results of the Bayesian network meta-analysis were similar to the results using the frequentist approach (Tables S8–S18).

## 4. Discussion

The results of this network meta-analysis, which looked at randomized controlled clinical trials to examine the efficacy, immunogenicity, and safety of various COVID-19 vaccines in the pre-delta era, provided information on these vaccinations. The efficacy of the 13 vaccines has been assessed through published trial results. The most effective vaccination type against COVID-19 infection, according to this study’s findings, is the mRNA vaccines, followed by the adenovirus-based and inactivated vaccines and finally the protein-subunit vaccines, which have the lowest efficacy. In contrast, the safest vaccine type, according to this study’s findings, is the inactivation vaccines, followed by the adenovirus-based and mRNA vaccines, which are the least safe. These results are similar to the trends of other previous studies [28,29,30]. In this study, the mRNA vaccines ranked best in terms of efficacy. However, mRNA vaccines showed the lowest safety profile when compared to other COVID-19 vaccine types in terms of local and systemic adverse events.

The adenovirus-based vaccines were also relatively efficacious. These vaccines induce antibody production by inserting antigen genes into a virus that has been treated so as not to harm the human body. They are then injected to enable cells to synthesize the antigens on their own [31]. Notably, the Gam-COVID-Vac vaccine had similar or better efficacy than the AZD1222 vaccine, which is produced using the same platform and has been approved by the EMA. The only difference is that different carriers are employed. The adenovirus antibodies against the vector are generated during the first vaccination, but if the same vector is used for the second vaccination, the antibody production does not increase [32]. The reason why AZD1222 has poor efficacy is because the vaccine’s efficacy also includes protection against the beta variant [33].

Inactivated vaccines ranked middle in terms of efficacy. In terms of safety, inactivation vaccines were the highest. It is significant to note that Sinopharm uses alum adjuvant, undoubtedly one of the most reactogenic adjuvants, which has been widely employed in various vaccine types available on the market. However, safety is extremely important, and patients were carefully monitored for the emergence of adverse drug events (ADEs) and vaccine-associated increased respiratory disease (VAERD). There was no indication of these occurrences in either the ongoing Phase III trial or the extended follow-ups. Additionally, the alum adjuvant is used in many different COVID-19 vaccines that are still in development, with no reports of VAERD. However, alum may lessen immunopathology when compared to COVID-19 vaccinations without adjuvant [34,35].

The protein-subunit vaccines received the lowest efficacy rank. These vaccines use specific protein fragments and polysaccharides that make up the pathogen’s shell or cell membrane as its main components [36]. Similar to inactivated vaccines, protein-subunit vaccines are generally safe [36]; however, the antigens are very small and lack the pathogen-associated molecular patterns required for antigen recognition by the host’s immune system, thereby reducing their immunogenicity [2]. Nevertheless, the protein-subunit-based platform is also being actively studied in the post-delta era.

Following the injection of the vaccines Ad26.COV2.S, AZD1222, BNT162b1, and mRNA-1273, a number of negative side effects have been documented [37,38]. The rates of thromboembolic events and myocarditis following COVID-19 infection are much higher than those after receiving the COVID-19 vaccines; however, it is crucial to note that these complications are incredibly rare [39,40]. Therefore, this cannot force persons who do not fall into high-risk categories to forego the chance to receive a preventive vaccine against a potentially deadly virus. Consequently, it is evident that the chance of experiencing post-vaccination thrombocytopenia is far lower than the risk of passing away or suffering serious side effects from SARS-CoV-2 infections, irrespective of the vaccine administered.

The inactivated vaccines and protein-subunit vaccines had a relatively low rate of ARs. These vaccines do not use whole parts of the pathogen but rather specific fragments of a disease-causing agent to stimulate the immune system, which might therefore be a relatively safe method compared with other platforms [2]. Most of the CoronaVac trials included in this study were conducted in China, in which the incidence of ARs was lower in the vaccinated group than in the placebo group. However, a trial conducted in Turkey found a higher incidence of ARs in the vaccinated group than in the placebo group [41]. The heterogeneity of AR reports needs to be considered when evaluating the safety of vaccines.

The mRNA and adenovirus-based vaccines showed a relatively high incidence of ARs compared with vaccines produced using other platforms, which is consistent with the findings of a previous meta-analysis [28,29,33,42]. According to a recent study analyzing real-world data on the safety of mRNA vaccinations, COVID-19 mRNA vaccinations were less dangerous than viral vector vaccines in terms of coagulation disorders, although inflammation-related AEs are less common with the viral vaccines [43]. Consideration should be given to the risk–benefit ratio of the vaccinations, and SAEs must be closely monitored and managed.

A UK-based study posited that although the effect of each vaccine was reduced due to the delta mutation, the relative effect size pattern was similar [44,45,46]. Contrarily, in the case of CoronaVac, as opposed to BNT162b2, the inducible neutralizing antibody dramatically decreased with time, leading to the increased chance of breakthrough infection [47]. There is no direct comparison data, but in the comparative analysis data on the effect of each vaccine on delta, those that are still effective against delta show the relative effect at the time of existing development [48]. Therefore, it is necessary to continue to follow up on the vaccines used in the pre-delta era.

RCT data and real-world evidence (RWE) are seen to be mutually beneficial [49]. A growing body of research indicates that properly executed RWE studies may be able to support regulatory decisions in the absence of RCT data. Further research may be required to better show the circumstances in which RWE analyses can reliably and consistently mirror the outcomes of RCTs and, more significantly, the circumstances in which they cannot. Regulators can then decide when to categorically accept RWE in place of an RCT after carefully examining the possibility of bias. Regulators may have to accept that the expense of expediting patient access to treatment involves a higher level of decision-making uncertainty than that of what they are accustomed to if studies based on RWE are ever to replace RCTs [49,50]. This study used RCTs rather than RWE for its analysis to further reduce uncertainty.

This study has several limitations. In terms of efficacy, there were large differences in the IgG measurement methods and results between the studies, and a number of the studies did not assess IgG response. Further well-planned direct comparison studies are needed to address this issue. This network meta-analysis analyzed the outcomes of clinical trials, and therefore the evaluation of indirect efficacy and information on COVID-19 vaccine breakthrough infections is limited. In the future, studies using RWE are expected to overcome these issues. In addition, this network meta-analysis should be cautiously interpreted in terms of neutralization because different trials utilized varied methodologies in evaluating these outcomes. In terms of safety, it was not possible to conduct a combined analysis of all studies because the AR reporting methods differed. These results should therefore be interpreted with caution. Furthermore, the included studies did not contain information on rare SAEs, such as vaccine-induced immune thrombotic thrombocytopenia, myocarditis, and pericarditis after post-approval administration [51]. Similar to other vaccines, the short- and long-term safety of COVID-19 vaccines should be continuously monitored using RWE to determine clear safety profiles. Despite these limitations, this study is the first indirect comparison using network meta-analysis to determine the relative efficacy and safety of COVID-19 vaccines and that of their platforms. The results of this study also address the controversy regarding the efficacy and safety of certain vaccines. Considering the debate over the efficacy and safety of certain vaccines due to the lack of direct head-to-head trials comparing numerous vaccines, the relative ranking of these vaccines and platforms provides possible evidence for this gray zone and is helpful for selecting vaccines or vaccine platform candidates.

In conclusion, the COVID-19 vaccinations that are currently in use are the most successful measure for limiting the pandemic. Future studies will be able to use the consolidated baseline data from this study to assess the efficacy and safety of the COVID-19 vaccines.

## Figures and Tables

**Table 1:** Characteristics of the vaccines included in the network meta-analysis.

Vaccine Type	Investigational Name	Company	Trade Name	Recommended	Adjuvant	Dosage
mRNA-based *	mRNA-1273	Moderna	Spikevax	Adults 18 and older	None	Two doses, 28 days apart
	BNT162b1	Pfizer/BioNTech	Comirnaty	Adults aged 16 and older (Emergency Use Authorization for ages 12–15)	None	Two doses, 21 days apart
Pro-Subunit	NVX-CoV2373	Novavax	Covovax	Ages 12–84	Matrix-M1	Two doses, 21 days apart
	ZF2001	Anhui Zhifei Longcom	Zifivax	Adults 18 and older	Aluminum hydroxide	Three doses, over a period of 2 months
	CoV2 preS dTM	Sanofi	-	Adults 18 and older	AF03	Two doses, 21 days apart
Adenovirus-based	Gam-COVID-Vac	Gamaleya	Sputnik V	Adults 18 and older	None	Two doses, 21 days apart
	Ad26.COV2.S	Johnson & Johnson	COVID-19 Vaccine Janssen	Adults 18 and older	None	Single shot
	AZD1222	Oxford/AstraZeneca	Covishield or Vaxzevria	Adults 18 and older	None	Two doses 28–84 days apart
	Ad5-nCoV	CanSino	Convidecia	Adults 18 and older	None	Single dose
Inactivated virus	MINHAI	SZKT	Kconvax	Adults 18 and older	Aluminum hydroxide	Two doses, 28 days apart
	BBIBP-CorV	Sinopharm	-	Adults 18 and older	Aluminum hydroxide	Two doses, 21 days apart
	WIV04	Sinopharm	-	Adults 18 and older	Aluminum hydroxide	Two doses, 21 days apart
	CoronaVac	Sinovac	CoronaVac	Adults 18 and older	Aluminum hydroxide	Two doses, 14–28 days apart

* The vaccine is being studied in children ages 5–11.

**Table 2:** Characteristics of the RCTs included in the network meta-analysis.

Study	Registered Trial Number	Phase	Intervention		Control	Patients			Ref.
			Treatment/Company	Dose (μg)		Total	Mean Age (years)	Female (%)	
Banden_2021	NCT04470427	Ⅲ	mRNA-1273/Moderna	100	Placebo	30,351	51.4	56	5
Chu_2021-1	NCT04405076	Ⅱ		50	Placebo	200	36.95	52	7
Chu_2021-2	NCT04405076	Ⅱ		100	Placebo	200	37.8	49.4	7
Chu_2021-3	NCT04405076	Ⅱ		50	Placebo	200	64.55	45	7
Chu_2021-4	NCT04405076	Ⅱ		100	Placebo	200	50.6	51.5	7
Formica_2021-1	NCT04368988	Ⅱ	NVX-CoV2373/Novavax	5	Placebo	513	51.55	51.05	23
Formica_2021-2	NCT04368988	Ⅱ		25	Placebo	514	52.15	50.55	23
Shinde_2021	NCT04533399	Ⅱ		5	Placebo	4382	32	42.6	25
Toback_2021	NCT04583995	Ⅲ		5	Placebo	14,039	56	48.4	24
Logunov_2021	NCT04530396	Ⅲ	Gam-COVID-Vac/Gamaleya	0.5 mL/dose	Placebo	19,866	45.3	48.5	8
Pan_2021-1	ChiCTR2000038804	Ι–Ⅱ	MINHAI/SZKT	5	Placebo	150	35.85	48.5	21
Pan_2021-2	ChiCTR2000038804	Ι–Ⅱ		10	Placebo	150	45.55	51	21
Pan_2021-3	ChiCTR2000038804	Ι–Ⅱ		5	Placebo	150	42.05	60	21
Pan_2021-4	ChiCTR2000038804	Ι–Ⅱ		10	Placebo	150	43.1	54	21
Polack_2021	NCT04368728	Ⅱ–Ⅲ	BNT162b1/Pfizer/BioNTech	30	Placebo	37,706	NA	55	1
Sadoff_2021	NCT04505722	Ⅲ	Ad26.COV2.S/Johnson & Johnson	0.5 mL/dose	Placebo	43,783	52	57	12
Sadoff_2021.01-1	NCT04436276	Ι–Ⅱ		5 × 10^10^ vp/mL	Placebo	244	35.75	51.5	11
Sadoff_2021.01-2	NCT04436276	Ι–Ⅱ		1 × 10^11^ vp/mL	Placebo	240	70.2	52.5	11
Sadoff_2021.01-3	NCT04436276	Ι–Ⅱ		5 × 10^10^ vp/mL	Placebo	242	69.75	50.5	11
Sadoff_2021.01-4	NCT04436276	Ι–Ⅱ		1 × 10^11^ vp/mL	Placebo	242	69.95	52	11
Xia_2020.10-1	ChiCTR2000032459	Ι–Ⅱ	BBIBP-CorV/Sinopharm	8	Placebo	112	60	45	20
Xia_2020.10-2	ChiCTR2000032459	Ι–Ⅱ		4	Placebo	112	54	51.5	20
Xia_2020.10-3	ChiCTR2000032459	Ι–Ⅱ		4	Placebo	112	55	42.5	20
Xia_2020.10-4	ChiCTR2000032459	Ι–Ⅱ		4	Placebo	112	57	50.5	20
Kaabi_2021-2	NCT04510207	Ⅲ		4	Placebo	25,463	36.1	15.35	19
Kaabi_2021-1	NCT04510207	Ⅲ	WIV04/Sinopharm_Wuhan	5	Placebo	25,480	36.15	15.6	19
Xia_2020-1	ChiCTR2000031809	Ι–Ⅱ		5	Placebo	112	35.1	52	18
Xia_2020-2	ChiCTR2000031809	Ι–Ⅱ		5	Placebo	112	35.1	48.5	18
Yang_2021-1	NCT04466085	Ι–Ⅱ	ZF2001/Anhui Zhifei Longcom	25	Placebo	300	56	48.5	26
Yang_2021-2	NCT04466085	Ι–Ⅱ		50	Placebo	300	58.5	51	26
Yang_2021-3	NCT04466085	Ι–Ⅱ		25	Placebo	300	43.05	52	26
Yang_2021-4	NCT04466085	Ι–Ⅱ		50	Placebo	300	43.3	54.5	26
Zhang_2021-1	NCT04352608	Ι–Ⅱ	CoronaVac/Sinovac	3	Placebo	180	42.8	56.65	16
Zhang_2021-2	NCT04352608	Ι–Ⅱ		6	Placebo	180	43	59.15	16
Zhang_2021-3	NCT04352608	Ι–Ⅱ		3	Placebo	180	42.9	48.75	16
Zhang_2021-4	NCT04352608	Ι–Ⅱ		6	Placebo	180	45.65	48.75	16
Wu_2021-1	NCT04383574	Ι–Ⅱ		1.5	Placebo	150	48.5	56	14
Wu_2021-2	NCT04383574	Ι–Ⅱ		3	Placebo	150	48.5	52	14
Wu_2021-3	NCT04383574	Ι–Ⅱ		6	Placebo	149	51	49.4	14
Bueno_2021	NCT04651790	Ⅱ		3	Placebo	310	NA	NA	15
Palacios_2021	NCT04456595	Ⅲ		3	Placebo	12,396	64.2	64.2	17
Zhu_2020-1	NCT04341389	Ⅱ	Ad5-nCoV/CanSino	1 × 10^11^ vp/mL	Placebo	379	39.6	49.9	9
Zhu_2020-2	NCT04341389	Ⅱ		5 × 10^10^ vp/mL	Placebo	255	39.45	49.9	9
Madhi_2021	NCT04444674	Ι–Ⅱ	AZD1222/ Oxford/ AstraZeneca	5 × 10^10^ vp/mL	Placebo	2021	NA	43.5	10
Goepfert_2021-1	NCT04537208	Ι–Ⅱ	CoV2 preS Dtm-AS03/Sanofi	1.3	Placebo	57	33.65	47	22
Goepfert_2021-2	NCT04537208	Ι–Ⅱ		1.3	Placebo	111	32.85	45	22
Goepfert_2021-3	NCT04537208	Ι–Ⅱ		2.6	Placebo	56	32.25	54.5	22
Goepfert_2021-4	NCT04537208	Ι–Ⅱ		2.6	Placebo	114	33.45	66.55	22
Goepfert_2021-5	NCT04537208	Ι–Ⅱ		1.3	Placebo	57	60.15	52.5	22
Goepfert_2021-6	NCT04537208	Ι–Ⅱ		1.3	Placebo	111	60.65	47	22
Goepfert_2021-7	NCT04537208	Ι–Ⅱ		2.6	Placebo	56	60.1	62.5	22
Goepfert_2021-8	NCT04537208	Ι–Ⅱ		2.6	Placebo	114	61.7	68	22

**Table 3:** Certainty of evidence evaluated with GRADE framework of efficacy.

Comparisons (vs. Placebo)	Study No.	Effect Size (95% CI)	Study Design	Grade	
Vaccine efficacy, RR					
mRNA-1273	1	0.05 (0.01, 0.47)	RCT	⊕⊕⊕○ Moderate	
NVX-CoV2373	2	0.23 (0.05, 1.07)	RCT	⊕⊕○○ Low	
BNT162b1	1	0.06 (0.01, 0.50)	RCT	⊕⊕⊕○ Moderate	
Gam-COVID-Vac	1	0.09 (0.01, 0.79)	RCT	⊕⊕⊕○ Moderate	
Ad26.COV2.S	1	0.32 (0.04, 2.61)	RCT	⊕⊕⊕○ Moderate	
AZD1222	1	0.25 (0.02, 2.84)	RCT	⊕⊕⊕○ Moderate	
BBIBP-CorV	1	0.27 (0.03, 2.24)	RCT	⊕⊕⊕○ Moderate	
WIV04	1	0.36 (0.04, 3.00)	RCT	⊕⊕⊕○ Moderate	
CoronaVac	1	0.50 (0.06, 4.07)	RCT	⊕⊕⊕○ Moderate	
Immunogenicity of neutralizing antibodies to live SARS-CoV-2, SMD					
mRNA-1273	1	1605.34 (1534.68, 1676.00)	RCT	⊕⊕⊕○ Moderate	
NVX-CoV2373	1	1360.28 (1019.39, 1701.18)	RCT	⊕⊕○○ Low	
Gam-COVID-Vac	1	42.91 (−14.77, 100.58)	RCT	⊕⊕⊕○ Moderate	
Ad26.COV2.S	1	223.28 (159.11, 287.44)	RCT	⊕⊕⊕○ Moderate	
BBIBP-CorV	2	142.29 (113.22, 171.36)	RCT	⊕⊕⊕○ Moderate	
WIV04	2	125.45 (86.83, 164.06)	RCT	⊕⊕⊕○ Moderate	
CoronaVac	1	35.01 (15.19, 54.83)	RCT	⊕⊕⊕○ Moderate	
ZF2001	1	13.97 (−13.92, 41.86)	RCT	⊕⊕⊕○ Moderate	
CoV2 preS Dtm-AS03/ Sanofi	1	22.15 (0.93, 43.36)	RCT	⊕⊕⊕○ Moderate	
Ad5-nCoV	1	18.90 (−20.53, 58.33)	RCT	⊕⊕⊕○ Moderate	
MINHAI	1	72.08 (43.25, 100.91)	RCT	⊕⊕⊕○ Moderate	
Immunogenicity of specific IgG, SMD					
MINHAI	1	1250.50 (942.25, 1558.74)	RCT	⊕⊕⊕○ Moderate	
NVX-CoV2373	1	1421.49 (426.76, 2416.22)	RCT	⊕⊕○○ Low	
Gam-COVID-Vac	1	8965.45 (7361.81, 10569.09)	RCT	⊕⊕⊕○ Moderate	
WIV04	1	143.96 (−234.55, 522.47)	RCT	⊕⊕⊕○ Moderate	
CoronaVac	1	1161.64 (907.67, 1415.62)	RCT	⊕⊕⊕○ Moderate	

High quality: High certainty that the actual effect closely matches the effect estimate, 4 of ⊕. Moderate quality: mediocre level of confidence in the impact estimate; the genuine effect is likely to be similar to the estimate, but there is a chance that it will be significantly different, 3 of ⊕. Low quality: little faith in the impact estimate; the actual effect could differ significantly from the estimated effect, 2 of ⊕.

## Data Availability

The data used for the findings of the current study are available upon request from the corresponding author.

## Supplementary Materials

The following are available online at www.mdpi.com/article/10.3390/vaccines10101572/s1, Figure S1. Network plot for indirect comparison of local and systemic AR, Unsolicited AR, and SAE. Figure S2. Forest plot of vaccine efficacy and immunogenicity of vaccine platform. Figure S3. Forest plot between vaccines of local and systemic AR, Unsolicited AR, and SAE of vaccine platform. Figure S4. Publication bias assessment of Vaccine efficacy and immunogenicity. Figure S5. Publication bias assessment between the vaccines of local and systemic AR, Unsolicited AR, and SAE. Figure S6. Risk of bias of include study. Figure S7. Meta-regression of vaccine efficacy and immunogenicity between vaccines. Figure S8. Meta-regression plot between the vaccines of local and systemic AR, Unsolicited AR, and SAE. Table S1. PRISMA NMA Checklist of Items to Include When Reporting a Systematic Review Involving a Network Meta-analysis. Table S2. Vaccines approved for more than one country and their publication data. Table S3. Definition of local AR, systemic AR, unsolicited AR, and serious adverse event. Table S4. Outcome measurement period or follow up duration for individual study. Table S5. network meta-analysis effect of efficacy and immunogenicity. Table S6. network meta-analysis effect of safety. Table S7. network meta-analysis heterogeneity. Table S8. Network estimated effect sizes (95% confidence interval) for Vaccine efficacy (Frequentist random effects model). Table S9. Network estimated effect sizes (95% confidence interval) for Neutralizing antibody responses to live SARS-CoV-2 (Frequentist random effects model). Table S10. Network estimated effect sizes (95% confidence interval) for Immunogenicity of specific and IgG antibody responses (Frequentist random effects model). Table S11. Network estimated effect sizes (95% confidence interval) for total dose of Any local AR (Frequentist random effects model). Table S12. Network estimated effect sizes (95% confidence interval) for total dose of Any systemic AR (Frequentist random effects model). Table S13. Network estimated effect sizes (95% confidence interval) for Any local AR of first vaccination (Frequentist random effects model). Table S14. Network estimated effect sizes (95% confidence interval) for Any local AR of second vaccination (Frequentist random effects model). Table S15. Network estimated effect sizes (95% confidence interval) for Any systemic AR of first vaccination (Frequentist random effects model). Table S16. Network estimated effect sizes (95% confidence interval) for Any systemic AR of second vaccination (Frequentist random effects model). Table S17. Network estimated effect sizes (95% confidence interval) for Unsolicited AR (Frequentist random effects model). Table S18. Network estimated effect sizes (95% confidence interval) for SAE (Frequentist random effects model). Table S19. Ranking of efficacy and immunogenicity. Table S20. Ranking of safety. Table S21. Ranking of safety continue. Table S22. Meta regression of efficacy. Table S23. Meta regression of Safety. Table S24. Deviance information criterion for model selection. Table S25. Network estimated effect sizes (95% confidence interval) for Vaccine efficacy (Bayesian random effects model). Table S26. Network estimated effect sizes (95% confidence interval) for Neutralizing antibody responses to live SARS-CoV-2 (Bayesian random effects model). Table S27. Network estimated effect sizes (95% confidence interval) for Any local AR of first vaccination (Bayesian random effects model). Table S28. Network estimated effect sizes (95% confidence interval) for Any local AR of second vaccination (Bayesian random effects model). Table S29. Network estimated effect sizes (95% confidence interval) for Any systemic AR of first vaccination (Bayesian random effects model). Table S30. Network estimated effect sizes (95% confidence interval) for Any systemic AR of second vaccination (Bayesian random effects model). Table S31. Network estimated effect sizes (95% confidence interval) for total dose of Any local AR (Bayesian random effects model). Table S32. Network estimated effect sizes (95% confidence interval) for total dose of Any systemic AR (Bayesian random effects model). Table S33. Network estimated effect sizes (95% confidence interval) for Unsolicited AR (Bayesian random effects model). Table S34. Network estimated effect sizes (95% confidence interval) for SAE (Bayesian random effects model). Table S35. Certainty of evidence evaluated with GRADE framework of safety.
