# Supplementary material for: Efficacy, Immunogenicity, and Safety of COVID-19 Vaccines in Randomized Control Trials in the Pre-Delta Era: A Systematic Review and Network Meta-Analysis
Source: Vaccines (Basel). 2022 Sep 20;10(10):1572. doi: 10.3390/vaccines10101572 (PMC9608197; doi:10.3390/vaccines10101572)
Supplement: Supplementary file 1 [file vaccines-10-01572-s001.zip › vaccines-1877552-supplementary.pdf]

Contents

Additional methods....4

Key term....4

Data synthesis and analysis....4

Supplementary figure....5

Figure S1. Network plot for indirect comparison of local and systemic AR, Unsolicited AR, and SAE....6

Figure S2. Forest plot of vaccine efficacy and immunogenicity of vaccine platform....7

Figure S3. Forest plot between vaccines of local and systemic AR, Unsolicited AR, and SAE of vaccine platform....8

Figure S4. Publication bias assessment of Vaccine efficacy and immunogenicity....9

Figure S5. Publication bias assessment between the vaccines of local and systemic AR, Unsolicited AR, and SAE....10

Figure S6. Risk of bias of include study....11

Figure S7. Meta-regression of vaccine efficacy and immunogenicity between vaccines....12

Figure S8. Meta-regression plot between the vaccines of local and systemic AR, Unsolicited AR, and SAE....13

Supplementary Tables....15

Table S1. PRISMA NMA Checklist of Items to Include When Reporting A Systematic Review Involving a Network Meta-analysis....15

Table S2. Vaccines approved for more than one country and their publication data....18

Table S3. Definination of local AR, systemic AR, unsolicited AR, and serious adverse event....20

Table S4. Outcome measurement priod or follow up duration for individual study.....21

Table S5. network meta-analysis effect of efficacy and immunogenicity.....25

Table S6. network meta-analysis effect of safety....26

Table S6. network meta-analysis effect of safety continue....27

Table S7. network meta-analysis heterogeneity....28

Table S8. Network estimated effect sizes (95% confidence interval) for Vaccine efficacy (Frequentist random effects model)....29

Table S9. Network estimated effect sizes (95% confidence interval) for Neutralizing antibody responses to live SARS-CoV-2 (Frequentist random effects model) 30

Table S10. Network estimated effect sizes (95% confidence interval) for Immunogenicity of specific and IgG antibody responses (Frequentist random effects model) 31

Table S11. Network estimated effect sizes (95% confidence interval) for total dose of Any local AR (Frequentist random effects model) 32

Table S12. Network estimated effect sizes (95% confidence interval) for total dose of Any systemic AR (Frequentist random effects model) 33

Table S13. Network estimated effect sizes (95% confidence interval) for Any local AR of first vaccination (Frequentist random effects model) 34

Table S14. Network estimated effect sizes (95% confidence interval) for Any local AR of second vaccination (Frequentist random effects model) 35

Table S15. Network estimated effect sizes (95% confidence interval) for Any systemic AR of first vaccination (Frequentist random effects model) 36

Table S16. Network estimated effect sizes (95% confidence interval) for Any systemic AR of second vaccination (Frequentist random effects model) 37

Table S17. Network estimated effect sizes (95% confidence interval) for Unsolicited AR (Frequentist random effects model) 38

Table S18. Network estimated effect sizes (95% confidence interval) for SAE (Frequentist random effects model) 39

Table S19. Ranking of efficacy and immunogenicity 40

Table S20. Ranking of safety 41

Table S21. Ranking of safety continue 42

Table S22. Meta regression of efficacy 43

Table S23. Meta regression of Safety 44

Table S24. Deviance information criterion for model selection 45

Table S25. Network estimated effect sizes (95% confidence interval) for Vaccine efficacy (Bayesian random effects model) 46

Table S26. Network estimated effect sizes (95% confidence interval) for Neutralizing antibody responses to live SARS-CoV-2 (Bayesian random effects model) 47

Table S27. Network estimated effect sizes (95% confidence interval) for Any local AR of first vaccination

(Bayesian random effects model) 48

Table S28. Network estimated effect sizes (95% confidence interval) for Any local AR of second vaccination (Bayesian random effects model) 49

Table S29. Network estimated effect sizes (95% confidence interval) for Any systemic AR of first vaccination (Bayesian random effects model) 50

Table S30. Network estimated effect sizes (95% confidence interval) for Any systemic AR of second vaccination (Bayesian random effects model) 51

Table S31. Network estimated effect sizes (95% confidence interval) for total dose of Any local AR (Bayesian random effects model) 52

Table S32. Network estimated effect sizes (95% confidence interval) for total dose of Any systemic AR (Bayesian random effects model) 53

Table S33. Network estimated effect sizes (95% confidence interval) for Unsolicited AR (Bayesian random effects model) 54

Table S34. Network estimated effect sizes (95% confidence interval) for SAE (Bayesian random effects model) 55

Table S35. Certainty of evidence evaluated with GRADE framework of safety 56

Table S35. Certainty of evidence evaluated with GRADE framework of safety (continue) 57

Reference 58

Additional methods

Key term

(("COVID-19"[MeSH Terms] OR "SARS-CoV-2"[MeSH Terms]) AND "Vaccines"[MeSH Terms]) AND (BBIBP-CorV OR BNT162b2 OR ((New Crown COVID-19) OR (SARS-COV-2 inactivated vaccine)) OR ((Gam-COVID-Vac) OR Gam-COVID-Vac) OR (CoronaVac OR (adsorbed COVID-19 inactivated vaccine) OR NVX-CoV2373 OR (AZD1222 OR (ChAdOx1 nCoV-19)) OR Ad5-nCoV OR (Ad26.COV2.S RR JNJ-78436735 OR Ad26COVS1) OR CoVLP OR mRNA-1273 OR COVISHIELD OR (RBD-dimer vaccine)OR(ZF2001)OR(CoV2 preS dTM)) AND (antibody))

Data synthesis and analysis

Frequentist and Bayesian NMA methods were used in accordance with the established methodology outlined by the National Institute of Health and Medical Sciences.[1] [2] This statistical method was conducting ITC[3,4] and is supported not only by the International Society for Pharmacoeconomics and Outcome Research (ISPRR) guidelines for indirect comparison and NMA, but also by the National Institute for Health and Clinical Excellence and Haute Autorité de Santé.[5,6] In the absence of RCTs demonstrating direct comparisons, the methodology of indirect comparison was useful for comparing treatment regimens[7] and has also been used in several other fields. For our analysis, This study was used the standard method of ITC, as described by Dias et al.[1] [2,8]

A frequentist random effects network meta-analysis was also performed using the NetMeta package on R.[9] P-score used to rank all therapies based on network estimates. In this study, higher values indicated better treatments. The model that was used was determined by the degree of heterogeneity, as low, moderate, and high, with upper limits of 25%, 50% and 75% for I^2, respectively.[9] Specific and IgG antibody responses only progressed frequentist analysis due to errors caused by too large variance in Bayesian analysis.

A Bayesian hierarchical network meta-analysis, which conducted heterogeneity between the included studies.[8] Analysis used the GeMTC package. Random effects were selected for outcome based on the deviance information criterion (DIC), using the model with the smallest value (Table S20). The probability that each vaccine was the most efficacy/safety was calculated by counting the proportion of iterations of the chain. Probability vaccine score was conducted surface under the cumulative ranking curve analysis (SUCRA). The SUCRA was that 1 is the best, and 0 is the worst.[10] All data were represented using Rstudio.

Supplementary figure

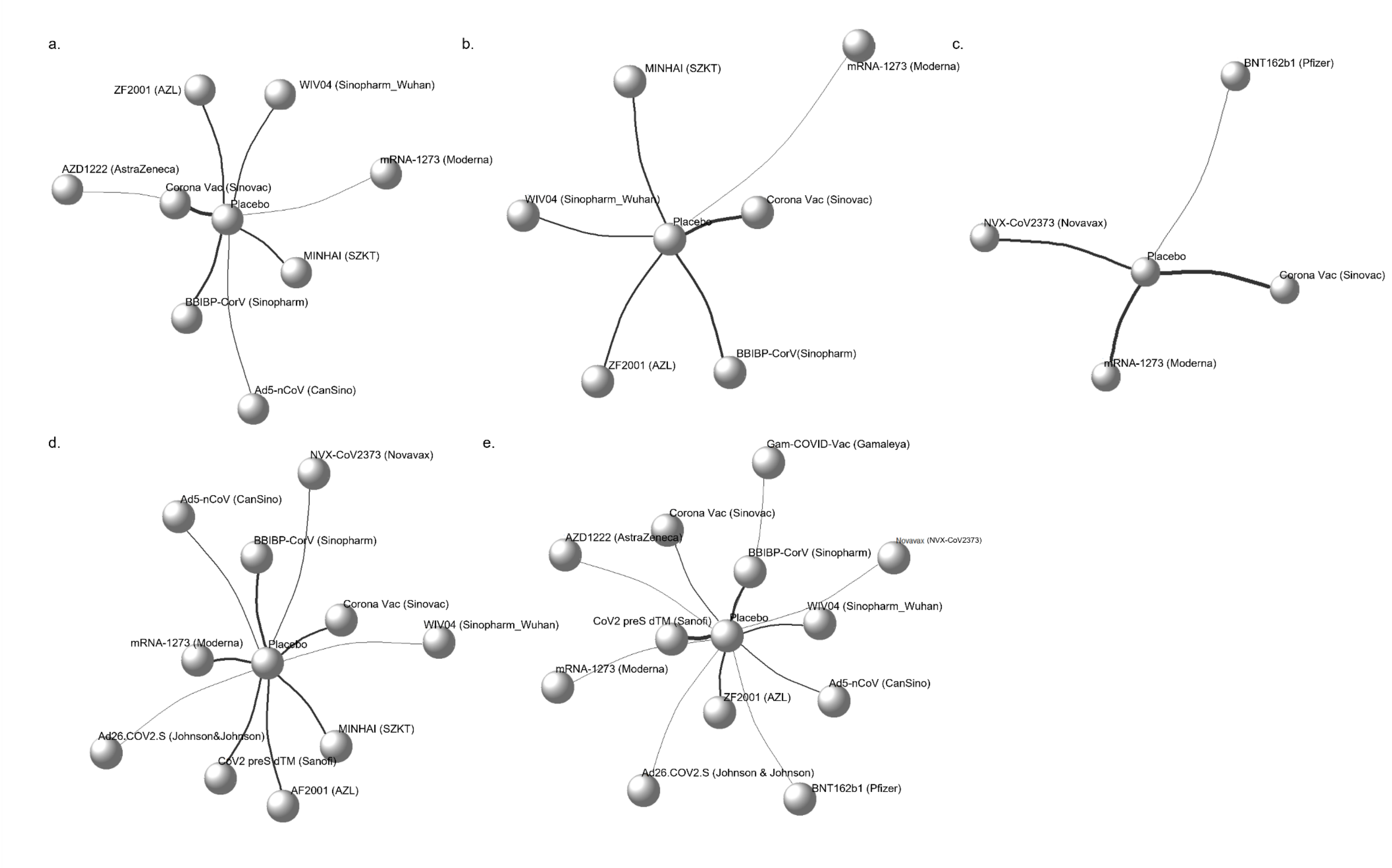

Figure S1. Network plot for indirect comparison of local and systemic AR, Unsolicited AR, and SAE

The size of the nodes is proportional to the number of subjects (sample size) randomized to receive the therapy. The width of the lines is proportional to the number of trials comparing each pair of treatments.

a. Any local adverse reaction of total dose; b. Any systemic adverse reaction of total dose; c. Any local and systemic adverse reaction of first and second vaccination; d. Unsolicited adverse reaction; e. Serious adverse events

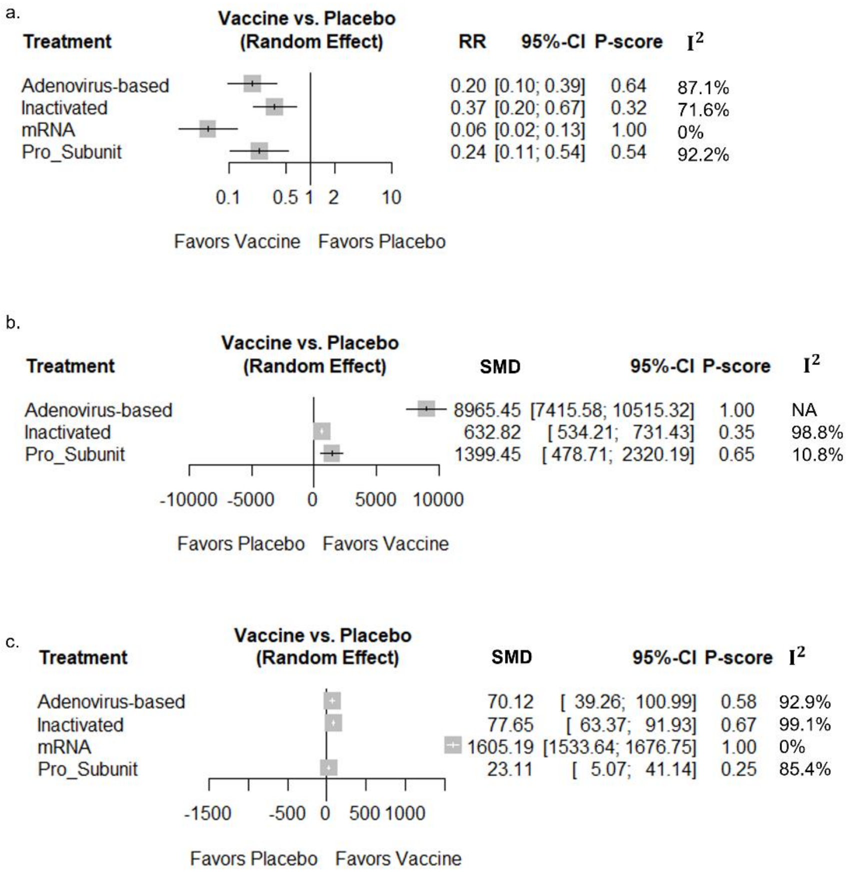

Figure S2. Forest plot of vaccine efficacy and immunogenicity of vaccine platform

a. Vaccine efficacy; b. Immunogenicity of specific and IgG antibody responses c. Neutralizing antibody responses to live SARS-CoV-2;

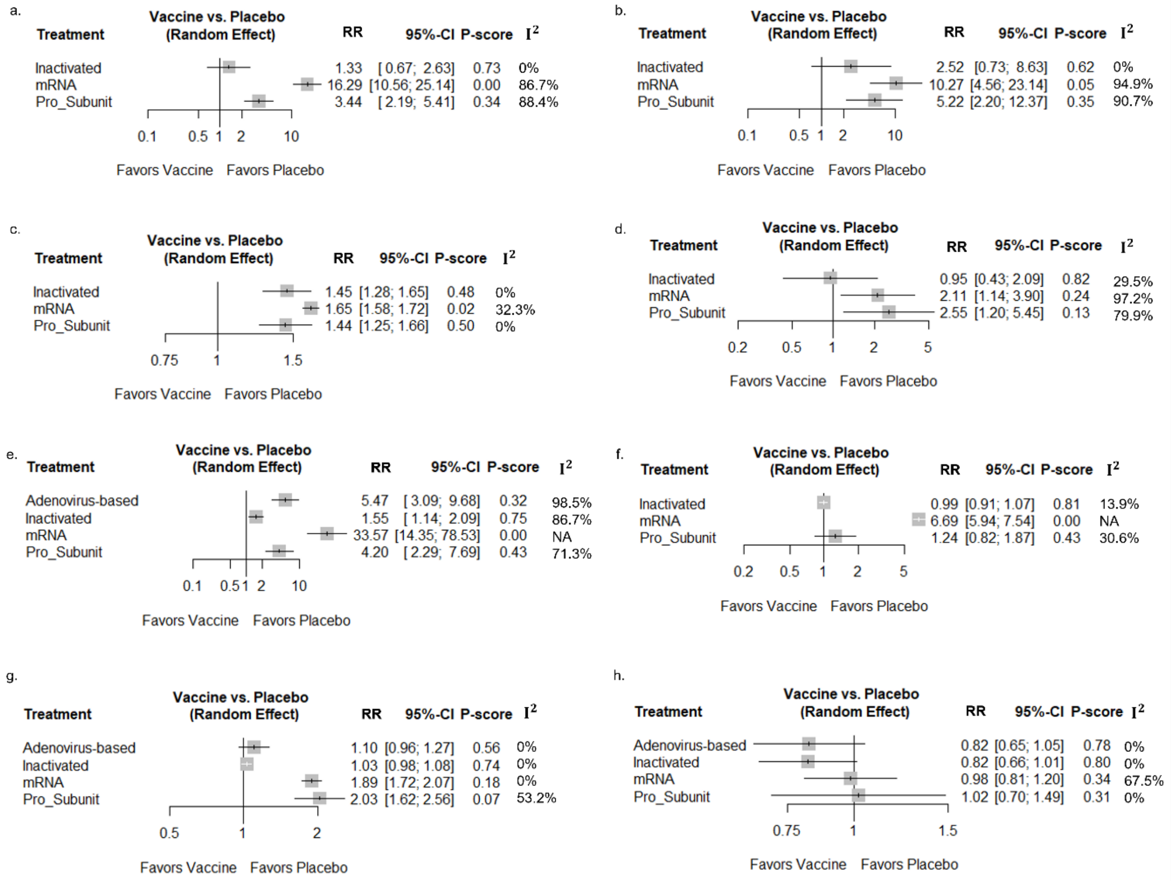

Figure S3. Forest plot between vaccines of local and systemic AR, Unsolicited AR, and SAE of vaccine platform

a. Any local adverse reaction of total vaccination; b. Any systemic adverse reaction of total vaccination; c. Any local adverse reaction of first vaccination; d. Any local adverse reaction of second vaccination; e. Any systemic adverse reaction of first vaccination; f. Any systemic adverse reaction of second vaccination; g. Unsolicited adverse reaction; h. Serious adverse event (SAE)

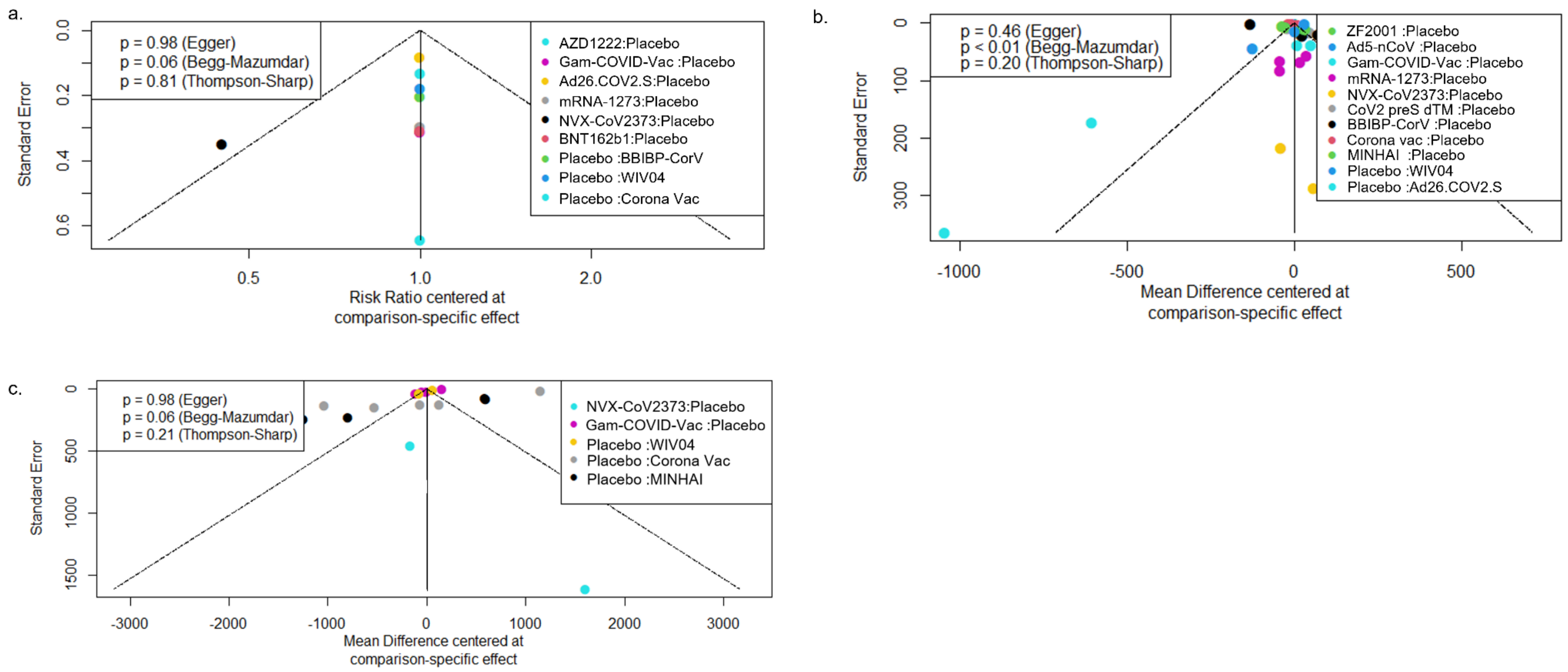

Figure S4. Publication bias assessment of Vaccine efficacy and immunogenicity

a. Vaccine efficacy; b. Neutralizing antibody responses to live SARS-CoV-2; c. Immunogenicity of specific and IgG antibody responses

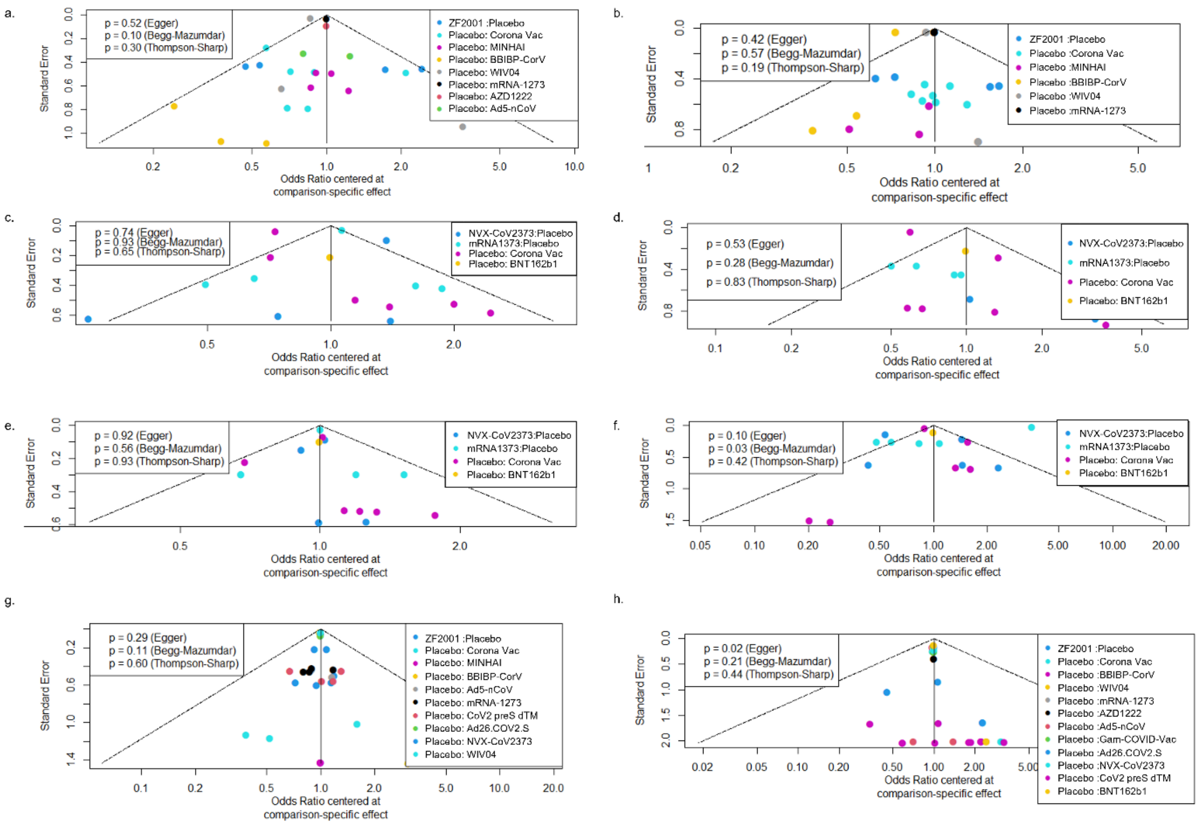

Figure S5. Publication bias assessment between the vaccines of local and systemic AR, Unsolicited AR, and SAE

a. Any local adverse reaction of total vaccination; b. Any systemic adverse reaction of total vaccination; c. Any local adverse reaction of first vaccination; d. Any local adverse reaction of second vaccination; e. Any systemic adverse reaction of first vaccination; f. Any systemic adverse reaction of second vaccination; g. Unsolicited adverse reaction; h. Serious adverse event (SAE)

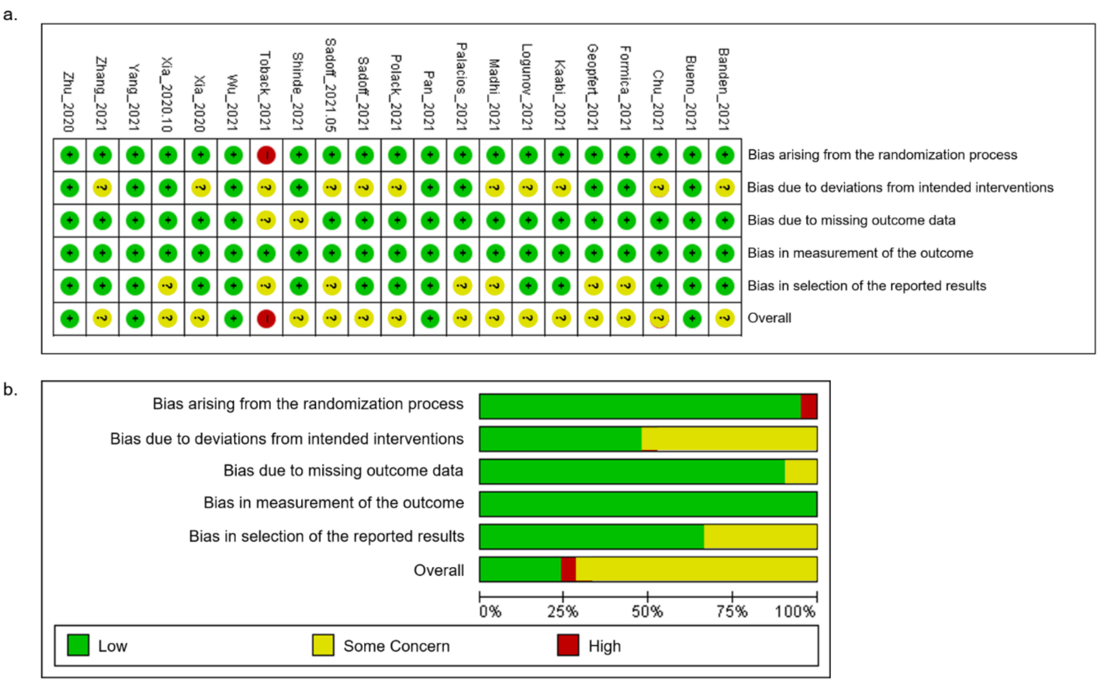

Figure S6. Risk of bias of include study

a. Risk of bias of summary; b. Risk of bias of graph

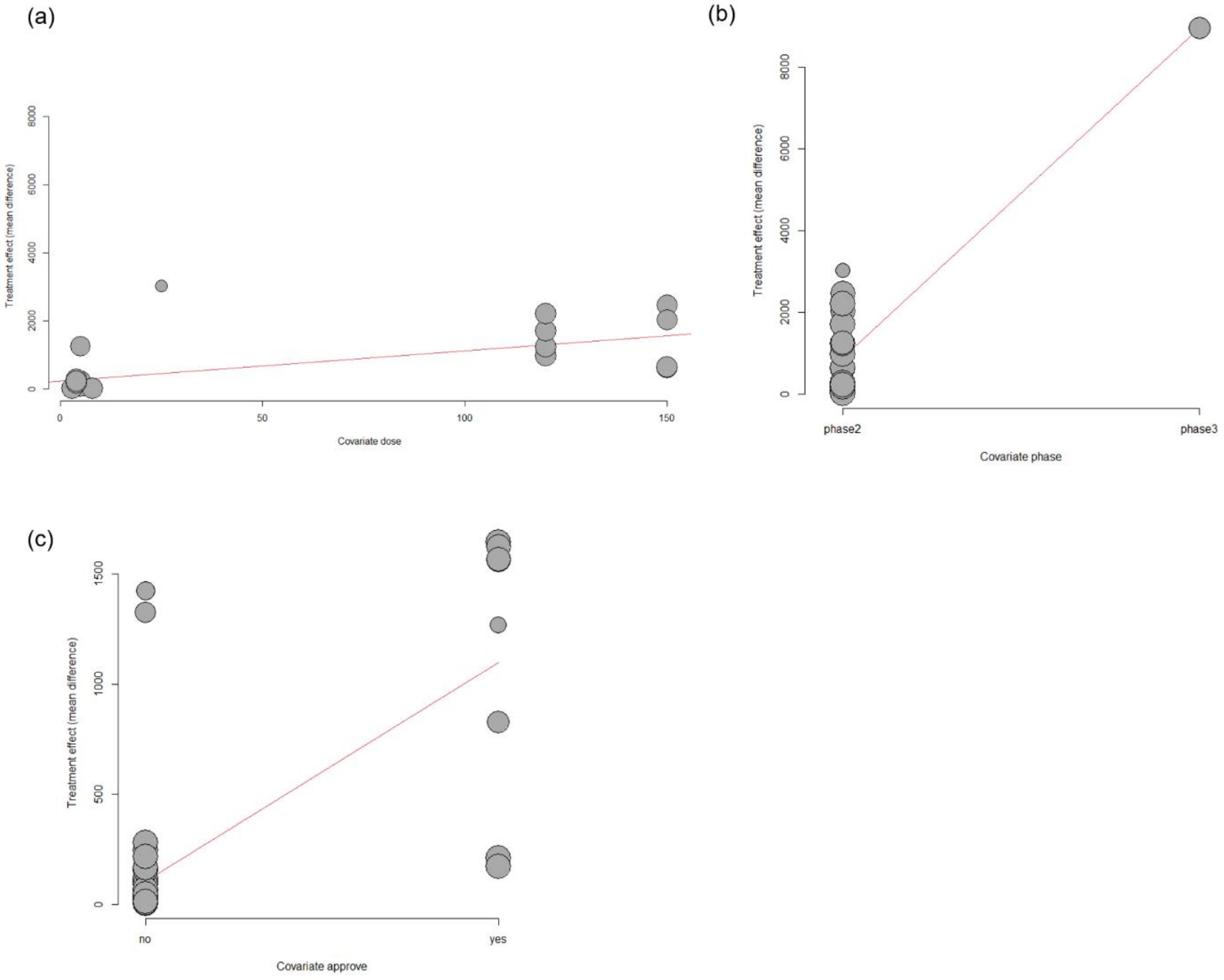

Figure S7. Meta-regression of vaccine efficacy and immunogenicity between vaccines

Bubble plot for covariate that shows significant statistical results in meta-regression.

a. Immunogenicity of specific and IgG antibody responses of covariate of dose; b. Immunogenicity of specific and IgG antibody responses of covariate of study phase; c. Neutralizing antibody responses to live SARS-CoV-2 of covariate EMA or FDA approval

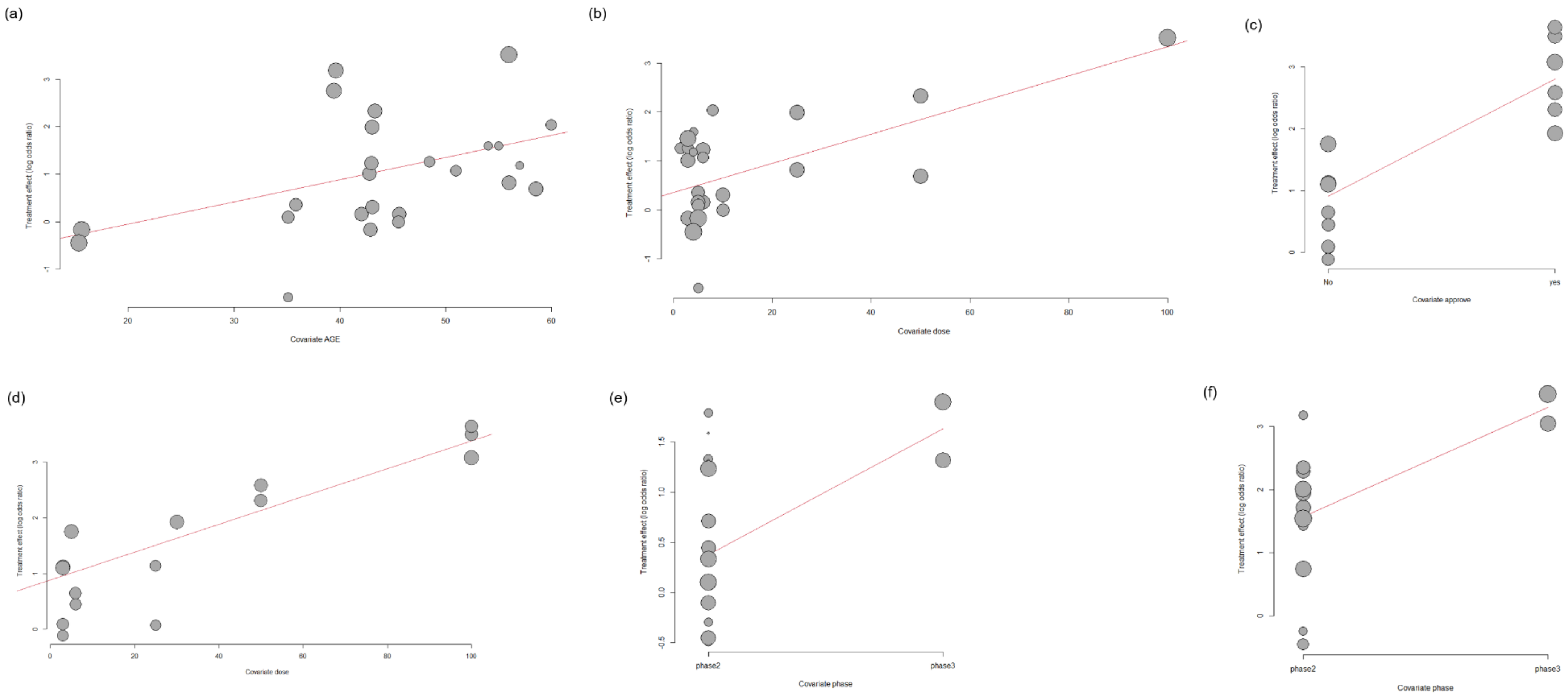

Figure S8. Meta-regression plot between the vaccines of local and systemic AR, Unsolicited AR, and SAE

Bubble plot for covariate that shows significant statistical results in meta-regression.

a. Any local AR of total dose of covariate AGE; b. Any local AR of total dose of covariate dose; c. Any local AR of first vaccination of covariate EMA or FDA approval; d. Any local AR of first vaccination of covariate dose; e. Any local AR of second vaccination of covariate study phase; f. Any systemic AR of second vaccination of covariate study phase

Supplementary Tables

Table S1. PRISMA NMA Checklist of Items to Include When Reporting A Systematic Review Involving a Network Meta-analysis

Section/Topic	Item #	Checklist Item	Reported on Page #
**TITLE**			
Title	1	Identify the report as a systematic review *incorporating a network meta-analysis (or related form of meta-analysis).*	1
**ABSTRACT**			
Structured summary	2	Provide a structured summary including, as applicable:<br>**Background:** main objectives<br>**Methods:** data sources; study eligibility criteria, participants, and interventions; study appraisal; and *synthesis methods, such as network meta-analysis.*<br>**Results:** number of studies and participants identified; summary estimates with corresponding confidence/credible intervals; *treatment rankings may also be discussed. Authors may choose to summarize pairwise comparisons against a chosen treatment included in their analyses for brevity.*<br>**Discussion/Conclusions:** limitations; conclusions and implications of findings.<br>**Other:** primary source of funding; systematic review registration number with registry name.	2
**INTRODUCTION**			
Rationale	3	Describe the rationale for the review in the context of what is already known, *including mention of why a network meta-analysis has been conducted.*_	3
Objectives	4	Provide an explicit statement of questions being addressed, with reference to participants, interventions, comparisons, outcomes, and study design (PICOS).	3
**METHODS**			
Protocol and registration	5	Indicate whether a review protocol exists and if and where it can be accessed (e.g., Web address); and, if available, provide registration information, including registration number.	4
Eligibility criteria	6	Specify study characteristics (e.g., PICOS, length of follow-up) and report characteristics (e.g., years considered, language, publication status) used as criteria for eligibility, giving rationale. *Clearly describe eligible treatments included in the treatment network, and note whether any have been clustered or merged into the same node (with justification).*_	4
Information sources	7	Describe all information sources (e.g., databases with dates of coverage, contact with study authors to identify additional studies) in the search and date last searched.	4
Search	8	Present full electronic search strategy for at least one database, including any limits used, such that it could be repeated.	4
Study selection	9	State the process for selecting studies (i.e., screening, eligibility, included in systematic review, and, if applicable, included in the meta-analysis).	4
Data collection process	10	Describe method of data extraction from reports (e.g., piloted forms, independently, in duplicate) and any processes for obtaining and confirming data from investigators.	4
Data items	11	List and define all variables for which data were sought (e.g., PICOS, funding sources) and any assumptions and simplifications made.	4
**Geometry of the network**	**S1**	Describe methods used to explore the geometry of the treatment network under study and potential biases related to it. This should include how the evidence base has been graphically summarized for presentation, and what characteristics were compiled and used	5

		to describe the evidence base to readers.	
Risk of bias within individual studies	12	Describe methods used for assessing risk of bias of individual studies (including specification of whether this was done at the study or outcome level), and how this information is to be used in any data synthesis.	4
Summary measures	13	State the principal summary measures (e.g., risk ratio, difference in means). *Also describe the use of additional summary measures assessed, such as treatment rankings and surface under the cumulative ranking curve (SUCRA) values, as well as modified approaches used to present summary findings from meta-analyses.*	4-5
Planned methods of analysis	14	Describe the methods of handling data and combining results of studies for each network meta-analysis. This should include, but not be limited to:<br>• *Handling of multi-arm trials;*<br>• *Selection of variance structure;*<br>• *Selection of prior distributions in Bayesian analyses; and*<br>• *Assessment of model fit.*	4-5
**Assessment of Inconsistency**	**S2**	Describe the statistical methods used to evaluate the agreement of direct and indirect evidence in the treatment network(s) studied. Describe efforts taken to address its presence when found.	NA
Risk of bias across studies	15	Specify any assessment of risk of bias that may affect the cumulative evidence (e.g., publication bias, selective reporting within studies).	4
Additional analyses	16	Describe methods of additional analyses if done, indicating which were pre-specified. This may include, but not be limited to, the following:<br>• Sensitivity or subgroup analyses;<br>• Meta-regression analyses;<br>• *Alternative formulations of the treatment network; and*<br>• *Use of alternative prior distributions for Bayesian analyses (if applicable).*_	4-5
**RESULTS†**			
Study selection	17	Give numbers of studies screened, assessed for eligibility, and included in the review, with reasons for exclusions at each stage, ideally with a flow diagram.	5
**Presentation of network structure**	**S3**	Provide a network graph of the included studies to enable visualization of the geometry of the treatment network.	5
**Summary of network geometry**	**S4**	Provide a brief overview of characteristics of the treatment network. This may include commentary on the abundance of trials and randomized patients for the different interventions and pairwise comparisons in the network, gaps of evidence in the treatment network, and potential biases reflected by the network structure.	5
Study characteristics	18	For each study, present characteristics for which data were extracted (e.g., study size, PICOS, follow-up period) and provide the citations.	5
Risk of bias within studies	19	Present data on risk of bias of each study and, if available, any outcome level assessment.	5
Results of individual studies	20	For all outcomes considered (benefits or harms), present, for each study: 1) simple summary data for each intervention group, and 2) effect estimates and confidence intervals. *Modified approaches may be needed to deal with information from larger networks.*	5-6
Synthesis of results	21	Present results of each meta-analysis done, including confidence/credible intervals. *In larger networks, authors may focus on comparisons versus a particular comparator (e.g. placebo or standard care), with full findings presented in an appendix. League tables and forest plots may be considered to summarize pairwise comparisons.* If additional summary measures were explored (such as treatment rankings), these should also be presented.	5-7
**Exploration for inconsistency**	**S5**	Describe results from investigations of inconsistency. This may include such information as measures of model fit to compare consistency and inconsistency models, *P* values from statistical tests, or summary of inconsistency estimates from different parts of the treatment network.	NA
Risk of bias across studies	22	Present results of any assessment of risk of bias across studies for the evidence base being studied.	5

Results of additional analyses	23	Give results of additional analyses, if done (e.g., sensitivity or subgroup analyses, meta-regression analyses, *alternative network geometries studied, alternative choice of prior distributions for Bayesian analyses,* and so forth).	7
**DISCUSSION**			
Summary of evidence	24	Summarize the main findings, including the strength of evidence for each main outcome; consider their relevance to key groups (e.g., healthcare providers, users, and policy-makers).	7
Limitations	25	Discuss limitations at study and outcome level (e.g., risk of bias), and at review level (e.g., incomplete retrieval of identified research, reporting bias). *Comment on the validity of the assumptions, such as transitivity and consistency. Comment on any concerns regarding network geometry (e.g., avoidance of certain comparisons).*	9
Conclusions	26	Provide a general interpretation of the results in the context of other evidence, and implications for future research.	9
**FUNDING**			
Funding	27	Describe sources of funding for the systematic review and other support (e.g., supply of data); role of funders for the systematic review. This should also include information regarding whether funding has been received from manufacturers of treatments in the network and/or whether some of the authors are content experts with professional conflicts of interest that could affect use of treatments in the network.	NA

PICOS = population, intervention, comparators, outcomes, study design.
* Text in italics indicateS wording specific to reporting of network meta-analyses that has been added to guidance from the PRISMA statement.
† Authors may wish to plan for use of appendices to present all relevant information in full detail for items in this section.

Table S2. Vaccines approved for more than one country and their publication data

Classification	Vaccine name (brand name, manufacturer)	Total number
Approval in more than one country despite publication	CIGB-66 (Abdala, CIGB), Aurora-CoV (EpiVacCorona, Vektor State Research Center of Virology and Biotechnology, Russia), ZF2001 (ZIFIVAX, AZL), mRNA-1273 (Spikevax, Moderna), AZD1222 (Covishield or Vaxzevria, AstraZeneca), Gam-COVID-Vac (Sputnik v, the Gamaleya Research Institute of Epidemiology and Microbiology, Russia), Ad26.COV2.S (COVID-19 Vaccine Janssen suspension for injection, Johnson & Johnson), Ad5-nCoV (Convidecia, CanSino), BBV152 (Covaxin, Bharat Biotech of India), QazCovid-in (QazVac, Research Institute for Biological Safety Problems, Kazakhstan), CoronaVac (Sinovac), BBIBP-CorV (Sinopharm Beijing), WIV04 (Sinopharm Wuhan), MVC-COV1901 (MVC COVID-19 vaccine, Medigen Vaccine Biologics Corporation in Taiwan, American company Dynavax Technologies and the U.S. National Institute of Health), BNT162b2 (Comirnaty, Pfizer & BioNTech), TAK-919 (Takeda Pharmaceutical Company) CoviVac (Chumakov Centre, Russia), Minhai COVID-19 vaccine (MINHAI, SZKT), KoviVac (Chumakov Center), ZyCoV-D (Zydus Cadila), Sputnik Layt (Sputnik Light, Gamaleya)	22
FDA or EMA approval despite publication	BNT162b2 (Comirnaty, Pfizer & BioNTech), mRNA-1273 (Spikevax, Moderna), AZD1222 (Covishield or Vaxzevria, AstraZeneca), Ad26.COV2.S (COVID-19 Vaccine Janssen suspension for injection, Janson&Janson)	4
Approval in more than one country for publication	Gam-COVID-Vac (Sputnik v, the Gamaleya Research Institute of Epidemiology and Microbiology, Russia), BBIBP-CorV (Sinopharm Beijing), WIV04 (Sinopharm Wuhan), CoronaVac (Sinovac), ZF2001 (ZIFIVAX, AZL), WIV04 (Sinopharm Wuhan), Ad5-nCoV	11

	(Convidecia, CanSino), Minhai COVID-19 vaccine (MINHAI, SZKT), BNT162b2 (Comirnaty, Pfizer & BioNTech), mRNA-1273 (Spikevax, Moderna), AZD1222 (Covishield or Vaxzevria, AstraZeneca), Ad26.COV2.S (COVID-19 Vaccine Janssen suspension for injection, Janson&Janson)	
No approval yet for publication	CoV2 preS dTM (Sanofi), NVX-CoV2373 (Novavax)	2

Table S3. Definination of local AR, systemic AR, unsolicited AR, and serious adverse event

Terms	Definition
Local adverse reaction	Reactogenicity included pain, tenderness, erythema, and swelling etc<br>occur where the shot was given. They are usually mild and occur within a few hours of the shot. Injections site pain, redness or swelling are most commonly reported local adverse reactions.
Systemic adverse reaction	Reactogenicity included fever, nausea or vomiting, headache, fatigue, malaise, myalgia, and arthralgia etc<br>include fever, headache, body aches, fatigue, etc. These reactions are usually mild and can last for several days.
Unsolicited adverse reaction	Preferred term and system organ class according to the Medical Dictionary for Regulatory Activities(MedDRA)
Serious adverse events	Serious adverse events were defined as any untoward medical occurrence that resulted in death, was life-threatening, required inpatient hospitalization or prolongation of existing hospitalization, or resulted in persistent disability/incapacity.

Table S4. Outcome measurement priod or follow up duration for individual study.

Study	Registered Trial number	Phase	Treatment/ company	Vaccine efficacy	Neutralizing antibodies	Specific IgG	AE	Follow up duration
Chu_2021	NCT04405076	II	mRNA-1273/Moderna	*	At baseline and on days 29, 43, and 57.	*	Unsolicited: 28 days after each injection	13-month follow-up to the end-of-study in this trial
Baden_2021	NCT04470427	III		At least 14 days after the second injection	*	*	Local: 7 days after each injection<br>Unsolicited: 28 days after each injection<br>Serious: from days 1 through days 759	Median follow up duration of at least 2 months after completion of 2 dose schedule
Formica_2021	NCT04368988	II	NVX-CoV2373 /Novavax	*	At days 0 (baseline), 21, and 35	At days 0 (baseline), 21, and 35	Unsolicited: through days 35 after first vaccination	-
Shinde_2021	NCT04533399	II		Within 7 days after second injection	*	*	-	Through 12 months after vaccination.
Toback_2021	NCT04583995	III		At least 7 days after the second vaccination	*	At baseline and on Day 35	Serious: within 21 days of study dose 1	-
Logunov_2021	NCT04530396	III	Gam-COVID-Vac/ Gamaleya	28 days after dose 1.	On the day of first vaccination and day 42	On the day of first vaccination and day 42.	Serious: at any time during the study	-
Pan_2021	ChiCTR2000038804	I-II	MINHAI/ SZKT	*	On days 14 and 28 after second vaccination	On days 14 and 28 after second vaccination	Local: 28 days after each injection<br>Systematic: 28 days	Until 12 months after second dose

							after each injection<br>Unsolicited: 28 days after each injection	
Polack_2021	NCT04368728	II-III	BNT162b1/ Pfizer/BioNTech	At least 7 days after the second dose	*	*	**	-
Sadoff_2021.01	NCT04436276	I-II	Ad26.COV2.S /Johnson&Johnson	*	On days 7, 29, and 71 after first vaccination	*	**	-
Sadoff_2021	NCT04505722	III		At least 14 and 28 days after administration	*	*	Unsolicited: for 28 days after administration<br>Serious: during the entire study	-
Xia_2020.10	ChiCTR2000032459	I-II	BBIBP-CorV/ Sinopharm	*	For the 8 µg schedule and the 4 µg days 0 and 28 schedule: on day 28 after the last inoculation<br>For the 4 µg days 0 and 14 and days 0 and 21 schedules: on day 14 and 28	*	Local: within 7 days after each injection<br>Systemic: within 7 days after each injection<br>Unsolicited: -<br>Serious: within 28 days post vaccination	-
Kaabi_2021	NCT04510207	III		At least 14 days after second dose.	Before first dose and on day 14 after the second dose.	*	Local: within 7 days after each injection<br>Unsolicited: within days 8~21 days after Serious:	-

							from the beginning of the first dose to 12 months after end of study	
Xia_2020	ChiCTR2000031809	I-II		*	14 days after each dose	14 days after each dose	Local: within 7 days after each injection<br>Systemic: for 7 days after each injection<br>Serious: within 12 months	12 months after the last inoculation
Yang_2021	NCT04466085	I-II	ZF2001/ Anhui Zhifei Longcom	*	At day 44 and 74	**	Local: within 7 days after each injection<br>Systemic: within 7 days after each injection<br>Unsolicited: -<br>Serious: upto 1 year after the first dose	At least 6 months after vaccination
Zhang_2021	NCT04352608	I-II	CoronaVac/ Sinovac	*	Days 0 and 14 cohort: at days 0 (baseline), 28, and 56<br>Days 0 and 28 cohort: on day 56	Days 0 and 14 cohort: at days 0 (baseline), 28, and 56<br>Days 0 and 28 cohort: on day 56	Local: within 28 days post vaccination<br>Systemic: within 28 days post vaccination<br>Unsolicited: within 28 days post vaccination	-
Wu_2021	NCT04383574	I-II		*	At day 28 after the second dose.	*	Local: within 28 days after each injection<br>Systemic: within 28 days after each vaccine	-

Bueno_2021	NCT04651790	II		*	At days 0 (baseline), 14, 28, and 42 after each injection	At days 0 (baseline), 14, 28, and 42 after each injection	Local: within 7 days after each injection<br>Serious: at any timepoint during the study	-
Palacios_2021	NCT04456595	III		At least 14 days after the second injection	*	*	Serious: -	Median follow-up of two months after the second dose
Zhu_2020-1	NCT04341389	II	Ad5-nCoV/ CanSino	*	At 28 days after each injection	**	Local: within 14 days after vaccination<br>Unsolicited: within 14 days after vaccination<br>Serious: up to 6 months	6 months post vaccination
Madhi_2021	NCT04444674	I-II	AZD1222/ Oxford/AstraZeneca	Onset more than 14 days after the second vaccination	*	*	Local: within the 7 days after an injection<br>Serious: -	-
Goepfert_2021	NCT04537208	I-II	CoV2 preS Dtm-AS03/ Sanofi	*	At days 1, 22, and 36 for each study group	**	Unsolicited: up to 21 days after each dose	Up to 12 months after the last study injection

Dash denotes that definition of the respective term used in our study was not clearly defined

*Included studies did not conducted the respective assays

**Reported data in the included studies could not be used in our analysis.

Table S5. network meta-analysis effect of efficacy and immunogenicity.

Data type	treatment	Frequentist approach<br><br>Effect (95% CI)_Random	Bayesian approach<br><br>Effect (95% CI)_Random
Vaccine efficacy	AZD1222	0.25 (0.02, 2.84)	0.22 (0.01, 15.00)
	Gam-COVID-Vac	0.09 (0.01, 0.79)	0.09 (0.00, 4.00)
	Ad26.COV2.S	0.32 (0.04, 2.61)	0.28 (0.01, 12.00)
	mRNA-1273	0.05 (0.01, 0.47)	0.06 (0.00, 2.20)
	NVX-CoV2373	0.23 (0.05, 1.07)	0.21 (0.02, 2.40)
	BNT162b1	0.06 (0.01, 0.50)	0.05 (0.00, 1.40)
	BBIBP-CorV	0.27 (0.03, 2.24)	0.26 (0.00, 5.90)
	WIV04	0.36 (0.04, 3.00)	0.38 (.002, 18.00)
	Corona Vac	0.50 (0.06, 4.07)	0.52 (0.01, 19.00)
	Placebo	Reference	Reference
Immunogenicity of specific and IgG antibody responses	Gam-COVID-Vac	8965.45 (7361.81, 10569.09)	-
	NVX-CoV2373	1421.49 (426.77, 2416.22)	-
	WIV04	143.96 (-234.55, 522.47)	-
	Corona Vac	1161.64 (907.67, 1415.62)	-
	MINHAI	1250.50 (942.25, 1558.74)	-
	Placebo	Reference	-
Neutralizing antibody responses to live SARS-CoV-2	ZF2001	13.97 (-13.92, 41.86)	14.00 (-6.30, 36.00)
	Ad5-nCoV	18.90 (-20.53, 58.33)	19.00 (-12.00, 51.00)
	Gam-COVID-Vac	42.91 (-14.77, 100.58)	43.00 (-4.50, 87.00)
	Ad26.COV2.S	223.28 (159.11, 287.44)	220.00 (160.00, 280.00)
	mRNA-1273	1605.34 (1534.68, 1676.00)	1600.00 (1500.00, 1700.00)
	NVX-CoV2373	1360.28 (1019.39, 1701.18)	1400.00 (1100.00, 1700.00)
	CoV2 preS dTM	22.15 (0.93, 43.36)	22.00 (6.00, 40.00)
	BBIBP-CorV	142.29 (113.22, 171.36)	150.00 (120.00, 190.00)
	WIV04	125.45 (86.83, 164.06)	120.00 (90.00, 150.00)
	Corona Vac	35.01 (15.19, 54.83)	34.00 (22.00, 49.00)
	MINHAI	72.08 (43.25, 100.91)	71.00 (45.00, 93.00)
	Placebo	Reference	Reference

Table S6. network meta-analysis effect of safety

Data type	treatment	Frequentist approach<br>Effect (95% CI)_Random	Bayesian approach<br>Effect (95% CI)_Random
Any local AR of total vaccination	AZD1222	1.04 (0.33, 3.23)	1.00 (0.25, 4.30)
	ZF2001	4.23 (2.08, 8.59)	4.50 (2.10, 10.00)
	Ad5-nCoV	19.52 (7.77, 49.00)	20.00 (6.80, 61.00)
	mRNA-1273	33.57 (10.90, 103.43)	32.00 (8.30, 150.00)
	BBIBP-CorV	1.86 (0.84, 4.09)	2.20 (1.00, 6.10)
	WIV04	0.72 (0.31, 1.70)	0.69 (0.29, 2.10)
	Corona Vac	2.44 (1.41, 4.23)	2.50 (1.40, 4.80)
	MINHAI	1.23 (0.56, 2.68)	1.30 (0.55, 3.00)
	Placebo	Reference	Reference
Any systemic AR of total vaccination	ZF2001	1.27 (0.77, 2.08)	1.30 (0.69, 2.40)
	mRNA-1273	6.69 (3.82, 11.71)	6.70 (2.80, 13.00)
	BBIBP-CorV	0.75 (0.49, 1.17)	0.77 (0.43, 1.30)
	WIV04	0.92 (0.55, 1.54)	0.91 (0.50, 1.70)
	Corona Vac	0.99 (0.64, 1.53)	0.85 (0.50, 1.40)
	MINHAI	1.37 (0.64, 2.90)	1.50 (0.71, 3.50)
	Placebo	Reference	Reference
Any local AR of first vaccination	mRNA-1273	20.25 (13.67, 30.01)	21.00 (13.00, 34.00)
	NVX-CoV2373	4.19 (2.51, 6.98)	3.10 (1.60, 6.40)
	BNT162b1	6.86 (3.20, 14.69)	6.60 (2.90, 17.00)
	Corona Vac	2.20 (1.50, 3.22)	2.30 (1.30, 3.60)
	Placebo	Reference	Reference
Any local AR of second vaccination	mRNA-1273	11.03 (4.61, 26.41)	12.00 (5.30, 35.00)
	NVX-CoV2373	7.34 (2.46, 21.93)	8.00 (2.60, 22.00)
	BNT162b1	7.44 (1.14, 48.50)	8.00 (0.98, 65.00)
	Corona Vac	2.80 (1.15, 6.85)	3.10 (1.20, 9.60)
	Placebo	Reference	Reference
Any systemic AR of first vaccination	mRNA-1273	1.66 (1.59, 1.74)	1.60 (1.30, 2.10)
	NVX-CoV2373	1.44 91.25, 1.66)	1.50 (1.10, 1.80)
	BNT162b1	1.46 (1.20, 1.78)	1.50 (1.00, 2.30)
	Corona Vac	1.45 (1.28, 1.65)	1.50 (1.10, 1.80)
	Placebo	Reference	Reference
Any systemic AR of second vaccination	mRNA-1273	1.88 (0.81, 4.35)	1.90 (0.89, 3.80)
	NVX-CoV2373	2.58 (1.04, 6.43)	2.60 (1.10, 5.40)
	BNT162b1	3.44 (0.55, 21.45)	3.40 (0.65, 13.00)
	Corona Vac	0.99 (0.39, 2.52)	1.10 (0.51, 2.60)
	Placebo	Reference	Reference
Unsolicated AR	ZF2001	0.74 (0.43, 1.28)	0.83 (0.44, 1.40)
	Ad5-nCoV	1.14 (0.59, 2.18)	1.10 (0.63, 2.10)
	Ad26.COV2.S	1.10 (0.95, 1.27)	1.10 (0.79, 1.50)
	mRNA-1273	1.89 (1.72, 2.07)	1.90 (1.50, 2.40)
	NVX-CoV2373	2.79 (2.08, 3.76)	2.90 (2.10, 3.90)
	CoV2 preS dTM	1.93 (1.19, 3.12)	2.00 (1.20, 3.60)
	BBIBP-CorV	1.01 (0.94, 1.08)	1.00 (0.72, 1.20)
	WIV04	1.05 (0.98, 1.12)	1.10 (0.80, 1.40)
	Corona Vac	0.79 (0.26, 2.37)	0.77 (0.38, 2.10)
	MINHAI	0.49 (0.12, 2.00)	0.46 (0.07, 1.70)
	Placebo	Reference	Reference

Table S6. network meta-analysis effect of safety continue

Data type	treatment	Frequentist approach<br>Effect (95% CI)_Random	Bayesian approach<br>Effect (95% CI)_Random
SAE	AZD1222	1.08 (0.50, 2.30)	1.92 (0.62, 3.39)
	ZF2001	1.31 (0.43, 3.98)	1.34 (0.58, 4.00)
	Ad5-nCoV	0.70 (0.04, 11.19)	0.73 (0.15, 12.20)
	Gam-COVID-Vac	0.64 (0.39, 1.05)	0.67 (0.40, 1.00)
	Ad26.COV2.S	0.86 (0.64, 1.16)	0.89 (0.86, 1.20)
	mRNA-1273	0.79 (0.58, 1.08)	0.80 (0.60, 1.26)
	NVX-CoV2373	0.98 (0.64, 1.49)	1.00 (0.94, 1.50)
	BNT162b1	1.14 (0.88, 1.47)	1.19 (0.80, 1.58)
	CoV2 preS dTM	1.09 (0.29, 4.11)	1.10 (0.30, 4.00)
	BBIBP-CorV	0.74 (0.53, 1.03)	0.78 (0.50, 1.10)
	WIV04	0.81 (0.58, 1.12)	0.83 (0.60, 1.24)
	Corona Vac	1.05 (0.64, 1.71)	1.03 (0.51, 1.65)
	Placebo	Reference	Reference

Table S7. network meta-analysis heterogeneity

Outcome	Model	Tau^2	Tau	I^2
Efficacy				
Vaccine efficacy	Random	1.133	1.064	92.2%
Immunogenicity of specific and IgG antibody responses	Random	73913.14	271.87	98.8%
Neutralizing antibody responses to live SARS-CoV-2	Random	805.64	28.38	98.1%
Safety				
Any local AR_vaccination1	Random	0.107	0.327	55.3%
Any local AR_vaccination2	Random	0.865	0.930	87.2%
Any systemic AR_vaccination1	Random	0.000	0.000	0.0%
Any systemic AR_vaccination2	Random	0.860	0.927	92.6%
Any local AR of total vaccination	Random	0.329	0.573	56.2%
Any systemic AR of total vaccination	Random	0.000	0.022	0.2%
Unsolicated AR	Random	0.000	0.000	0%
SAE	Random	0.000	0.000	0%

Table S8. Network estimated effect sizes (95% confidence interval) for Vaccine efficacy (Frequentist random effects model)

The columns present the row drug class compared to the column vaccine class. The rows present the row vaccine class compared to the column vaccine class. The effect estimates are expressed as risk ratio and 95% confidence intervals. For example, Vaccine efficacy with AZD1222 compared to Gam-COVID-Vac is 1.01 (95% confidence interval -2.25 to 4.28).

ADZ1222									
1.01 (-2.25, 4.28)	Gam-COVID-Vac								
-0.26 (-3.47, 2.95)	-1.27 (-4.29, 1.74)	AD26.COV2.S							
1.53 (-1.73, 4.79)	0.51 (-2.55, 3.58)	1.79 (-1.22, 4.80)	mRNA-1273						
0.08 (-2.81, 2.96)	-0.94 (-3.60, 1.73)	0.34 (-2.26, 2.93)	-1.45 (-4.10, 1.21)	NVX-CoV2373					
1.47 (-1.79, 4.74)	0.46 (-2.61, 3.53)	1.73 (-1.28, 4.75)	-0.05 (-3.12, 3.01)	1.39 (-1.27, 4.06)	BNT162b2				
-1.39 (-3.83, 1.05)	-2.40 (-4.58, -0.23)	-1.13 (-3.22, 0.96)	-2.92 (-5.08, -0.75)	-1.47 (-3.01, 0.07)	-2.86 (-5.04, -0.69)	Placebo			
-0.07 (-3.31, 3.16)	-1.09 (-4.12, 1.95)	0.19 (-2.79, 3.17)	-1.60 (-4.63, 1.43)	-0.15 (-2.77, 2.47)	-1.55 (-4.58, 1.49)	1.32 (-0.80, 3.44)	BBIBP-CorV		
-0.38 (-3.60, 2.85)	-1.39 (-4.42, 1.65)	-0.12 (-3.09, 2.86)	-1.90 (-4.93, 1.12)	-0.45 (-3.07, 2.16)	-1.85 (-4.88, 1.18)	1.02 (-1.10, 3.13)	-0.30 (-3.30, 2.69)	WIV04	
-0.69 (-3.91, 2.52)	-1.71 (-4.73, 1.32)	-0.43 (-3.40, 2.53)	-2.22 (-5.24, 0.80)	-0.77 (-3.37, 1.83)	-2.17 (-5.19, 0.86)	0.70 (-1.40, 2.80)	-0.62 (-3.61, 2.37)	-0.32 (-3.30, 2.66)	Corona Vac

Table S9. Network estimated effect sizes (95% confidence interval) for Neutralizing antibody responses to live SARS-CoV-2 (Frequentist random effects model)

The columns present the row drug class compared to the column vaccine class. The rows present the row vaccine class compared to the column vaccine class. The effect estimates are expressed as mean difference and 95% confidence intervals. For example, Neutralizing antibody responses to live SARS-CoV-2 with AZL compared to CanSino is -4.93 (95% confidence interval -53.23 to 43.37).

ZF2001											
-4.93<br>(-53.23, 43.37)	Ad5-nCoV										
-28.94<br>(-93.00, 35.13)	-24.01<br>(-93.87, 45.86)	Gam-COVID-Vac									
-209.31<br>(-279.27, -139.34)	-204.37<br>(-279.69, -139.06)	-180.37<br>(-266.64, -94.09)	AD26.COV2.S								
-1591.37<br>(-1667.33, -1515.40)	-1586.44<br>(-1667.35, -1505.52)	-1562.43<br>(-1653.64, -1471.22)	-1382.06<br>(-1477.51, -1286.61)	mRNA-1273							
-1346.31<br>(-1688.35, 1-1004.28)	-1341.38<br>(-1684.56, -998.21)	-1317.38<br>(-1663.12, -971.63)	-1137.01<br>(-1483.89, -790.12)	245.05<br>(-103.09, 593.20)	NVX-CoV2373						
13.97<br>(-13.92, 41.86)	18.90<br>(-20.53, 58.33)	42.91<br>(-14.77, 100.58)	223.28<br>(159.11, 287.44)	1605.34<br>(1534.68, 1676.00)	1360.28<br>(1019.39, 1701.18)	Placebo					
-8.17<br>(-43.22, 26.87)	-3.24<br>(-48.02, 41.53)	20.76<br>(-40.69, 82.22)	201.13<br>(133.55, 268.71)	1583.19<br>(1509.41, 1656.97)	1338.14<br>(996.58, 1679.70)	-22.15<br>(-43.36, -0.93)	CoV2 preS dTM				
-128.32<br>(-168.61, -88.03)	-123.39<br>(-172.37, -74.40)	-99.38<br>(-163.97, -34.79)	80.99<br>(10.54, 151.43)	1463.05<br>(1386.64, 1539.45)	1218.00<br>(875.86, 1560.13)	-142.29<br>(-171.36, -113.22)	-120.14<br>(-156.13, -84.15)	BBIBP-CorV			
-111.47<br>(-159.11, -63.84)	-106.54<br>(-161.73, -51.35)	-82.54<br>(-151.95, -13.13)	97.83<br>(22.94, 172.72)	1479.89<br>(1399.37, 1560.42)	1234.84<br>(891.76, 1577.92)	-125.45<br>(-164.06, -86.83)	-103.30<br>(-149.36, -59.24)	16.84<br>(-31.49, 65.18)	WIV04		
-21.04<br>(-55.26, 13.18)	-16.11<br>(-60.24, 28.02)	7.90<br>(-53.09, 68.88)	188.26<br>(121.11, 255.42)	1570.33<br>(1496.94, 1643.71)	1325.27<br>(983.80, 1666.75)	-35.01<br>(-54.83, -15.19)	-12.87<br>(-41.90, 16.17)	107.28<br>(72.09, 142.46)	90.43<br>(47.02, 133.84)	Corona Vac	
-58.11<br>(-98.22, -17.99)	-53.18<br>(-102.02, -4.33)	-29.17<br>(-93.65, 35.31)	151.20<br>(80.85, 221.54)	1533.26<br>(1456.94, 1609.57)	1288.21<br>(946.09, 1630.32)	-72.08<br>(-100.91, -43.25)	-49.93<br>(-85.73, -14.14)	70.21<br>(29.27, 111.15)	53.37<br>(5.17, 101.56)	-37.07<br>(-72.05, -2.08)	MINHAI

Table S10. Network estimated effect sizes (95% confidence interval) for Immunogenicity of specific and IgG antibody responses (Frequentist random effects model)

The columns present the row drug class compared to the column vaccine class. The rows present the row vaccine class compared to the column vaccine class. The effect estimates are expressed as mean difference and 95% confidence intervals. For example, Immunogenicity of specific and IgG antibody responses with Gam-COVID-Vac compared to NVX-CoV2373 is 7543.96 (95% confidence interval 5656.85 to 9431.06).

Gam-COVID-Vac					
7543.96<br>(5656.85, 9431.06)	NVX-CoV2373				
8965.45<br>(7361.81, 10569.09)	1421.49<br>(426.76, 2416.22)	Placebo			
8821.49<br>(7173.78, 10469.20)	1277.54<br>(213.23, 2341.85)	-143.96<br>(-522.47, 234.55)	WIV04		
7803.81<br>(6180.18, 9427.44)	259.85<br>(-766.79, 1286.49)	-1161.64<br>(-1415.62, -907.67)	-1017.69<br>(-1473.51, -561.86)	Corona Vac	
7714.95<br>(6081.95, 9347.95)	171.00<br>(-870.40, 1212.39)	-1250.50<br>(-1558.74, -942.25)	-1106.54<br>(-1594.69, -618.39)	-88.85<br>(-488.25, 310.54)	MINHAI

Table S11. Network estimated effect sizes (95% confidence interval) for total dose of Any local AR (Frequentist random effects model)

The columns present the row drug class compared to the column vaccine class. The rows present the row vaccine class compared to the column vaccine class. The effect estimates are expressed as odds ratio and 95% confidence intervals. For example, the mean difference for any systemic AR of total dose with AZD1222 compared to ZF2001 is -1.41 (95% confidence interval -2.75 to -0.07).

AZD1222								
-1.41 (-2.75, -0.07)	ZF2001							
-2.94 (-4.40, -1.47)	-1.53 (-2.69, -0.37)	Ad5-nCoV						
-3.48 (-5.08, -1.88)	-2.07 (-3.40, -0.74)	-0.54 (-2.00, 0.91)	mRNA-1273					
0.04 (-1.10, 1.17)	1.44 (0.73, 2.15)	2.97 (2.05, 3.89)	3.51 (2.39, 4.64)	Placebo				
-0.58 (-1.97, 0.80)	0.82 (-0.24, 1.88)	2.35 (1.14, 3.56)	2.89 (1.52, 4.27)	-0.62 (-1.41, 0.17)	BBIBP-CorV			
0.36 (-1.06, 1.78)	1.76 (0.65, 2.87)	3.29 (2.04, 4.55)	3.84 (2.42, 5.25)	0.32 (-0.53, 1.18)	0.94 (-0.22, 2.11)	WIV04		
-0.86 (-2.12, 0.41)	0.55 (-0.35, 1.44)	2.08 (1.01, 3.15)	2.62 (1.37, 3.87)	-0.89 (-1.44, -0.34)	-0.27 (-1.24, 0.69)	-1.22 (-2.23, -0.20)	Corona Vac	
-0.17 (-1.55, 1.21)	1.23 (0.18, 2.29)	2.76 (1.56, 3.97)	3.31 (1.94, 4.68)	-0.21 (-0.99, 0.57)	0.41 (-0.70, 1.52)	-0.53 (-1.69, 0.63)	0.69 (-0.27, 1.64)	MINHAI

Table S12. Network estimated effect sizes (95% confidence interval) for total dose of Any systemic AR (Frequentist random effects model)

The columns present the row drug class compared to the column vaccine class. The rows present the row vaccine class compared to the column vaccine class. The effect estimates are expressed as odds ratio and 95% confidence intervals. For example, the mean difference for any systemic AR of total dose with ZF2001 compared to mRNA-1273 is -1.66 (95% confidence interval -2.41 to -0.92).

ZF2001						
-1.66<br>(-2.41, -0.92)	mRNA-1273					
0.24<br>(-0.26, 0.73)	1.90<br>(1.34, 2.46)	Placebo				
0.52<br>(-0.14, 1.18)	2.19<br>(1.48, 2.90)	0.29<br>(-0.15, 0.72)	BBIBP-CorV			
0.32<br>(-0.39, 1.03)	1.98<br>(1.22, 2.74)	0.08<br>(-0.43, 0.60)	-0.20<br>(-0.88, 0.47)	WIV04		
0.25<br>(-0.41, 0.91)	1.91<br>(1.20, 2.62)	0.01<br>(-0.43, 0.45)	-0.27<br>(-0.89, 0.35)	-0.07<br>(-0.75, 0.60)	Corona Vac	
-0.08<br>(-0.98, 0.83)	1.59<br>(0.65, 2.53)	-0.31<br>(-1.07, 0.44)	-0.60<br>(-1.47, 0.27)	-0.40<br>(-1.31, 0.52)	-0.32<br>(-1.19, 0.55)	MINHAI

Table S13. Network estimated effect sizes (95% confidence interval) for Any local AR of first vaccination (Frequentist random effects model)

The columns present the row drug class compared to the column vaccine class. The rows present the row vaccine class compared to the column vaccine class. The effect estimates are expressed as odds ratio and 95% confidence intervals. For example, the mean difference for Any local AR of first vaccination with mRNA-1273 compared to NVX-CoV2373 is 1.58 (95% confidence interval 0.93 to 2.22).

mRNA-1273				
1.58 (0.93, 2.22)	NVX-CoV2373			
1.08 (0.23, 1.94)	-0.49 (-1.41, 0.42)	BNT162b2		
3.01 (2.61, 3.40)	1.43 (0.92, 1.94)	1.93 (1.16, 2.69)	Placebo	
2.22 (1.67, 2.77)	0.65 (0.01, 1.28)	1.14 (0.29, -0.40)	-0.79 (-1.17, -0.40)	Corona Vac

Table S14. Network estimated effect sizes (95% confidence interval) for Any local AR of second vaccination (Frequentist random effects model)

The columns present the row drug class compared to the column vaccine class. The rows present the row vaccine class compared to the column vaccine class. The effect estimates are expressed as odds ratio and 95% confidence intervals. For example, the mean difference for Any local AR of second vaccination with mRNA-1273 compared to NVX-CoV2373 is 0.41 (95% confidence interval -0.99 to 1.81).

mRNA-1273				
0.41 (-0.99, 1.81)	NVX-CoV2373			
0.39 (-1.67, 2.46)	-0.01 (-2.18, 2.16)	BNT162b2		
2.40 (1.53, 3.27)	1.99 (0.90, 3.09)	2.01 (0.13, 3.88)	Placebo	
1.37 (0.12, 2.62)	0.96 (-0.45, 2.37)	0.98 (-1.10, 3.05)	-1.03 (-1.92, -0.14)	Corona Vac

Table S15. Network estimated effect sizes (95% confidence interval) for Any systemic AR of first vaccination (Frequentist random effects model)

The columns present the row drug class compared to the column vaccine class. The rows present the row vaccine class compared to the column vaccine class. The effect estimates are expressed as odds ratio and 95% confidence intervals. For example, the mean difference for any systemic AR of first vaccination with mRNA-1273 compared to NVX-CoV2373 is 0.14 (95% confidence interval -0.01 to 0.29).

mRNA-1273				
0.14<br>(-0.01, 0.29)	NVX-CoV2373			
0.13<br>(-0.08, 0.33)	-0.02<br>(-0.26, 0.23)	BNT162b2		
0.51<br>(0.46, 0.55)	0.37<br>(0.22, 0.51)	0.38<br>(0.18, 0.58)	Placebo	
0.13<br>(0.00, 0.27)	-0.01<br>(-0.20, 0.18)	0.01<br>(-0.23, 0.24)	-0.37<br>(-0.50, -0.24)	Corona Vac

Table S16. Network estimated effect sizes (95% confidence interval) for Any systemic AR of second vaccination (Frequentist random effects model)

The columns present the row drug class compared to the column vaccine class. The rows present the row vaccine class compared to the column vaccine class. The effect estimates are expressed as odds ratio and 95% confidence intervals. For example, the mean difference for any systemic AR of second vaccination with mRNA-1273 compared to NVX-CoV2373 is -0.32 (95% confidence interval -1.56 to 0.92).

mRNA-1273				
-0.32 (-1.56, 0.92)	NVX-CoV2373			
-0.61 (-2.62, 1.41)	-0.29 (-2.33, 1.76)	BNT162b2		
0.63 (-0.21, 1.47)	0.95 (0.04, 1.86)	1.24 (-0.59, 3.07)	Placebo	
0.64 (-0.62, 1.90)	0.96 (-0.35, 2.27)	1.25 (-0.81, 3.30)	0.01 (-0.92, 0.95)	Corona Vac

Table S17. Network estimated effect sizes (95% confidence interval) for Unsolicited AR (Frequentist random effects model)

The columns present the row drug class compared to the column vaccine class. The rows present the row vaccine class compared to the column vaccine class. The effect estimates are expressed as odds ratio and 95% confidence intervals. For example, the mean difference for unsolicited AR with ZF2001 compared to Ad5-nCoV is -0.43 (95% confidence interval -1.28 to 0.42).

ZF2001										
-0.43<br>(-1.28, 0.42)	Ad5-nCoV									
-0.40<br>(-0.96, 0.17)	0.03<br>(-0.64, 0.70)	AD26.COV2.S								
-0.93<br>(-1.49, -0.38)	-0.51<br>(-1.16, 0.15)	-0.54<br>(-0.71, -0.37)	mRNA-1273							
-1.33<br>(-1.95, -0.71)	-0.90<br>(-1.61, -0.18)	-0.93<br>(-1.26, -0.60)	-0.39<br>(-0.70, -0.08)	NVX-CoV2373						
-0.30<br>(-0.84, 0.25)	0.13<br>(-0.52, 0.78)	0.10<br>(-0.05, 0.24)	0.63<br>(0.54, 0.73)	1.03<br>(0.73, 1.32)	Placebo					
-0.96<br>(-1.69, -0.23)	-0.53<br>(-1.34, 0.28)	-0.56<br>(-1.07, -0.06)	-0.02<br>(-0.52, 0.47)	0.37<br>(-0.20, 0.94)	-0.66<br>(-1.14, -0.17)	CoV2 preS dTM				
-0.31<br>(-0.85, 0.24)	-0.12<br>(-0.53, 0.78)	0.09<br>(-0.07, 0.25)	0.63<br>(0.51, 0.74)	1.02<br>(0.72, 1.32)	-0.01<br>(-0.07, 0.06)	0.65<br>(0.16, 1.14)	BBIBP-CorV			
-0.35<br>(-0.89, 0.20)	0.08<br>(-0.57, 0.73)	0.05<br>(-0.11, 0.21)	0.59<br>(0.47, 0.70)	0.98<br>(0.68, 1.28)	-0.05<br>(-0.11, 0.02)	0.61<br>(0.12, 1.10)	-0.04<br>(-0.13, 0.05)	WIV04		
-0.06<br>(-1.29, 1.17)	0.37<br>(-0.91, 1.65)	034<br>(-0.78, 1.45)	0.87<br>(-0.23, 1.98)	1.27<br>(0.12, 2.41)	0.24<br>(-0.86, 1.34)	0.90<br>(-0.31, 2.10)	0.25<br>(-0.86, 1.35)	0.29<br>(-0.82, 1.39)	Corona Vac	
0.40<br>(-1.09, 1.90)	0.83<br>(-0.71, 2.37)	0.80<br>(-0.60, 2.20)	1.34<br>(-0.06, 2.74)	1.73<br>(0.30, 3.16)	0.70<br>(-0.69, 2.10)	1.36<br>(-0.12, 2.84)	0.71<br>(-0.69, 2.11)	0.75<br>(-0.65, 2.15)	0.46<br>(-1.32, 2.24)	MINHAI

Table S18. Network estimated effect sizes (95% confidence interval) for SAE (Frequentist random effects model)

The columns present the row drug class compared to the column vaccine class. The rows present the row vaccine class compared to the column vaccine class. The effect estimates are expressed as odds ratio and 95% confidence intervals. For example, the mean difference for SAE with AZD1222 compared to ZF2001 is -0.15 (95% confidence interval -1.37 to 1.07).

AZD1222												
-0.15 (-1.37, 1.07)	ZF2001											
0.44 (-1.67, 2.55)	0.59 (-1.60, 2.77)	Ad5-nCoV										
0.53 (-0.38, 1.44)	0.68 (-0.40, 1.76)	0.09 (-1.93, 2.12)	Gam-COVID-Vac									
0.22 (-0.60, 1.03)	0.37 (-0.63, 1.37)	-0.22 (-2.21, 1.77)	-0.31 (-0.89, 0.26)	AD26.COV2.S								
0.31 (-0.51, 1.13)	0.46 (-0.55, 1.47)	-0.13 (-2.12, 1.86)	-0.22 (-0.34, 0.52)	0.09 (-0.34, 0.52)	mRNA-1273							
0.10 (-0.77, 0.96)	0.25 (-0.80, 1.29)	-0.34 (-2.35, 1.67)	-0.44 (-1.08, 0.21)	-0.12 (-0.63, 0.39)	-0.21 (-0.73, 0.31)	NVX-CoV2373						
-0.05 (-0.85, 0.75)	0.10 (-0.90, 1.09)	-0.49 (-2.47, 1.49)	-0.58 (-1.14, -0.03)	-0.27 (-0.66, 0.12)	-0.36 (-0.77, 0.04)	-0.15 (-0.64, 0.34)	BNT162b2					
0.07 (-0.69, 0.83)	0.22 (-0.74, 1.18)	-0.36 (-2.33, 1.60)	-0.46 (-0.95, 0.04)	-0.14 (-0.44, 0.15)	-0.23 (-0.55, 0.08)	-0.02 (-0.44, 0.39)	0.13 (-0.13, 0.38)	Placebo				
0.14 (-1.09, 1.37)	0.29 (-1.08, 1.65)	-0.30 (-2.49, 1.89)	-0.39 (-1.48, 0.70)	-0.08 (-1.09, 0.93)	-0.17 (-1.19, 0.85)	0.04 (-1.01, 1.10)	0.19 (-0.81, 1.19)	0.06 (-0.91, 1.03)	CoV2 preS dTM			
0.40 (-0.43, 1.23)	0.55 (-0.47, 1.56)	-0.04 (-2.03, 1.95)	-0.13 (-0.73, 0.46)	0.18 (-0.26, 0.62)	0.09 (-0.36, 0.54)	0.30 (-0.23, 0.83)	0.45 (0.04, 0.87)	0.32 (0.00, 0.65)	0.26 (-0.76, 1.28)	BBIBP-CorV		
0.30 (-0.53, 1.12)	0.44 (-0.57, 1.46)	-0.14 (-2.13, 1.85)	-0.24 (-0.83, 0.36)	0.08 (-0.36, 0.51)	-0.01 (-0.46, 0.44)	0.20 (-0.33, 0.73)	0.35 (-0.06, 0.76)	0.22 (-0.10, 0.55)	0.16 (-0.87, 1.18)	-0.10 (-0.56, 0.36)	WIV04	
0.05 (-0.86, 0.95)	0.19 (-0.88, 1.27)	-0.39 (-2.42, 1.63)	-0.49 (-1.18, 0.21)	-0.17 (-0.74, 0.39)	-0.26 (-0.84, 0.31)	-0.05 (-0.69, 0.59)	0.10 (-0.45, 0.65)	-0.03 (-0.51, 0.46)	-0.09 (-1.18, 0.99)	-0.35 (-0.94, 0.23)	-0.25 (-0.83, 0.33)	Corona Vac

Table S19. Ranking of efficacy and immunogenicity

Vaccine efficacy	Bayesian		Frequentist	
	SUCRA	Ranking	P-SCRRE	Ranking
mRNA-1273	0.7714	1	0.8178	1
BNT162b1	0.7633	2	0.8077	2
Gam-COVID-Vac	0.6606	3	0.7176	3
NVX-CoV2373	0.5175	4	0.5031	4
AZD1222	0.4939	5	0.4803	5
BBIBP-CorV	0.4867	6	0.4626	6
Ad26.COV.S	0.4536	7	0.4158	7
WIV04	0.3734	8	0.3875	8
Corona Vac	0.3222	9	0.3106	9
Placebo	0.1544	10	0.0970	10

Immunogenicity of specific and IgG antibody responses	Bayesian		Frequentist	
	SUCRA	Ranking	P-SCRRE	Ranking
Gam-COVID-Vac	-	-	1.0000	1
NVX-CoV2373	-	-	0.7172	2
MINHAI	-	-	0.6643	3
Corona Vac	-	-	0.6153	4
WIV04	-	-	0.2084	5
Placebo	-	-	0.0515	6

Neutralizing antibody responses to live SARS-CoV-2	Bayesian		Frequentist	
	SUCRA	Ranking	P-SCRRE	Ranking
mRNA-1273	0.9916	1	0.9924	1
NVX-CoV2373	0.9175	2	0.9167	2
Ad26.COV.S	0.8161	3	0.8166	3
BBIBP-CorV	0.7207	4	0.7057	4
WIV04	0.6136	5	0.6571	5
MINHAI	0.5327	6	0.5261	6
Gam-COVID-Vac	0.3880	7	0.3668	7
Corona Vac	0.3659	8	0.3522	8
CoV2 preS dTM	0.2345	9	0.2420	9
Ad5-nCoV	0.2064	10	0.2139	10
ZF2001	0.1625	11	0.1714	11
Placebo	0.0205	12	0.0391	12

Table S20. Ranking of safety

Any local AR of total vaccination	Bayesian		Frequentist	
	SUCRA	Ranking	P-SCRRE	Ranking
WIV04	0.8966	1	0.9012	1
Placebo	0.8081	2	0.7984	2
AZD1222	0.7609	3	0.7584	3
MINHAI	0.6984	4	0.6958	4
BBIBP-CorV	0.4781	5	0.5252	5
Corona Vac	0.4425	6	0.4192	6
ZF2001	0.2834	7	0.2757	7
Ad5-nCoV	0.0941	8	0.0966	8
mRNA-1273	0.0378	9	0.0292	9

Any systemic AR of total vaccination	Bayesian		Frequentist	
	SUCRA	Ranking	P-SCRRE	Ranking
BBIBP-CorV	0.8329	1	0.8554	1
WIV04	0.7346	2	0.6200	2
Corona Vac	0.6696	3	0.5323	3
Placebo	0.5713	4	0.5152	4
ZF2001	0.3671	5	0.2441	5
MINHAI	0.3204	6	0.2330	6
mRNA-1273	0.0042	7	0.0001	7

Any local AR of first vaccination	Bayesian		Frequentist	
	SUCRA	Ranking	P-SCRRE	Ranking
Placebo	0.9981	1	1.0000	1
Corona Vac	0.6894	2	0.7430	2
NVX-CoV2373	0.5388	3	0.4694	3
BNT162b2	0.2713	4	0.2860	4
mRNA-1273	0.0025	5	0.0017	5

Any local AR of second vaccination	Bayesian		Frequentist	
	SUCRA	Ranking	P-SCRRE	Ranking
Placebo	0.9888	1	0.9925	1
Corona Vac	0.6681	2	0.6817	2
BNT162b2	0.3513	3	0.3342	3
NVX-CoV2373	0.3256	4	0.3279	4
mRNA-1273	0.1663	5	0.1637	5

Table S21. Ranking of safety continue

Any systemicAR of first vaccination	Bayesian		Frequentist	
	SUCRA	Ranking	P-SCRRE	Ranking
Placebo	0.9938	1	1.0000	1
NVX-CoV2373	0.4738	2	0.5132	2
Corona Vac	0.4613	3	0.4918	3
BNT162b2	0.4200	4	0.4532	4
mRNA-1273	0.1513	5	0.0418	5

Any systemic AR of second vaccination	Bayesian		Frequentist	
	SUCRA	Ranking	P-SCRRE	Ranking
Placebo	0.8656	1	0.8267	1
Corona Vac	0.7756	2	0.7897	2
mRNA-1273	0.4356	3	0.4114	3
NVX-CoV2373	0.2438	4	0.2527	4
BNT162b2	0.1794	5	0.2195	5

Unsolicated AR	Bayesian		Frequentist	
	SUCRA	Ranking	P-SCRRE	Ranking
MINHAI	0.9245	1	0.8577	1
ZF2001	0.7175	2	0.8198	2
Corona Vac	0.7075	3	0.7086	3
Placebo	0.6353	4	0.6693	4
BBIBP-CorV	0.3605	5	0.6362	5
WIV04	0.5378	6	0.5147	6
Ad5-nCoV	0.5360	7	0.4998	7
Ad26.COV2.S	0.4795	8	0.4509	8
mRNA-1273	0.1645	9	0.1690	9
CoV2 preS dTM	0.1500	10	0.1602	10
NVX-CoV2373	0.0170	11	0.0139	11

Serious adverse event	Bayesian		Frequentist	
	SUCRA	Ranking	P-SCRRE	Ranking
Gam-COVID-Vac	0.7438	1	0.8259	1
BBIBP-CorV	0.6921	2	0.7499	2
mRNA-1273	0.6225	3	0.6589	3
WIV04	0.5969	4	0.6434	4
Ad5-nCoV	0.5346	5	0.6031	5
Ad26.COV2.S	0.5302	6	0.5550	6
CoV2 preS dTM	0.5079	7	0.4734	7
NVX-CoV2373	0.4321	8	0.4096	8
Corona Vac	0.4138	9	0.3631	9
Placebo	0.4042	10	0.3574	10
AZD1222	0.3931	11	0.3558	11
ZF2001	0.3202	12	0.2792	12
BNT162b2	0.3088	13	0.2254	13

Table S22. Meta regression of efficacy

Co-Variate	Regression Coefficient, 95%CI	P value
Vaccine efficacy (Overall)		
AGE	-0.015 (-0.088, 0.058)	0.625
SEX (Female %)	-0.014 (-0.053, 0.026)	0.447
Phase	-0.806 (-2.463, 0.852)	0.295
Approve (yes)	-0.728 (-1.981, 0.525)	0.217
Dose	-0.020 (-0.042, 0.002)	0.069
Immunogenicity of specific and IgG antibody response (Overall)		
AGE	-23.770 (-169.529, 121.988)	0.733
SEX (Female %)	29.805 (-214.351, 273.962)	0.798
Phase	8077.965 (-5616.239, 10539.691)	<0.0001
*Adjuvant (yes)	635.134 (-2980.226, 4250.493)	0.715
Dose	8.881 (4.054, 13.708)	0.001
Immunogenicity of specific and IgG antibody response (Vaccine platform_inactivated)		
AGE	-29.025 (-92.538, 33.942)	0.333
SEX (Female %)	85.724 (-8.492, 179.940)	0.071
Dose	9.665 (5.058, 14.272)	0.001
Neutralizing antibody to live SARS-CoV-2 (Overall)		
AGE	0.265 (-14.945, 15.474)	0.972
SEX (Female %)	-1.806 (-19.210, 15.598)	0.835
Phase	-204.430 (-824.844, 415.983)	0.510
Approve (yes)	990.467 (711.086, 1269.848)	<0.0001
Adjuvant (yes)	-88.228 (-427.285, 250.830)	0.602
Dose	-0.586 (-2.346, 1.174)	0.504
Neutralizing antibody to live SARS-CoV-2 (Vaccine platform_adenovirus based)		
AGE	-8.598 (-38.556, 21.360)	0.494
SEX (Female %)	190.218 (-63.312, 443.748)	0.112
Phase	-289.034 (-1476.626, 898.557)	0.559
Approve (yes)	433.348 (-199.627, 1066.323)	0.139
Neutralizing antibody to live SARS-CoV-2 (Vaccine platform_inactivated)		
AGE	0.450 (-4.753, 5.652)	0.858
SEX (Female %)	-1.527 (-4.637, 1.582)	0.315
Phase	38.030 (-83.060, 159.120)	0.518
*Adjuvant (yes)	90.256 (6.803, 173.710)	0.036
Dose	-0.353 (-0.785, 0.078)	0.103
AGE	0.450 (-4.753, 5.652)	0.858
Neutralizing antibody to live SARS-CoV-2 (Vaccine platform_protein subunit)		
AGE	-0.066 (-0.709, 0.577)	0.828
SEX (Female %)	0.127 (-1.555, 1.809)	0.872
Adjuvant (yes)	-0.816 (-16.233, 14.601)	0.910
Dose	0.003 (-0.049, 0.055)	0.907

* Adjuvant (yes) was used Matrix-M1

Table S23. Meta regression of Safety

Co-Variate	Regression Coefficient, 95%CI	P value
Any local AR of total dose (Overall)		
AGE	0.047 (0.008, 0.086)	0.020
SEX (Female %)	0.058 (-0.019, 0.136)	0.132
Approve (yes)	0.851 (-0.755, 2.457)	0.286
Dose	0.030 (0.017, 0.043)	<0.0001
Any local AR of total dose (Vaccine platform_inactivated)		
AGE	0.034 (0.020, 0.047)	<0.0001
SEX (Female %)	0.048 (0.019, 0.077)	0.003
Dose	-0.049 (-0.231, 0.134)	<0.0001
Any local AR of first vaccination		
AGE	0.022 (-0.047, 0.091)	0.502
SEX (Female %)	-0.007 (-0.139, 0.125)	0.913
Phase	0.925 (-0.901, 2.750)	0.296
Approve (yes)	1.889 (1.174, 2.604)	<0.0001
Adjuvant (yes)	-0.059 (-1.788, 1.671)	0.943
Dose	0.025 (0.017, 0.033)	<0.0001
Any local AR of second vaccination		
AGE	0.031 (-0.025, 0.088)	0.254
SEX (Female %)	0.037 (-0.073, 0.148)	0.482
Phase	1.739 (0.654, 2.823)	0.004
Approve (yes)	0.911 (-0.166, 1.99)	0.091
Adjuvant (yes)	1.148 (-0.290, 2.586)	0.109
Dose	0.012 (-0.002, 0.026)	0.085
Any systemic AR of second vaccination		
AGE	-0.008 (-0.048, 0.028)	0.629
SEX (Female %)	0.002 (-0.068, 0.071)	0.962
Phase	1.257 (0.418, 2.096)	0.006
Approve (yes)	.0295 (-0.528, 1.117)	0.457
Adjuvant (yes)	0.656 (-0.247, 1.559)	0.143
Dose	0.005 (-0.006, 0.016)	0.332

* Adjuvant (yes) was used Matrix-M1

Table S24. Deviance information criterion for model selection

Outcome	Model	Dbar*	pD**	Deviance Information Criterion (DIC)
Efficacy				
Vaccine efficacy	Random	20.709	20.162	40.871
Neutralizing antibody responses to live SARS-CoV-2	Random	37.539	32.732	70.271
Safety				
Any local AR of total vaccination	Random	53.961	41.324	95.285
Any systemic AR of total vaccination	Random	40.248	28.590	68.838
Any local AR of first vaccination	Random	33.594	26.379	59.973
Any local AR of second vaccination	Random	24.573	22.780	47.353
Any systemic AR of first vaccination	Random	26.208	20.775	46.982
Any systemic AR of second vaccination	Random	28.842	24.407	53.249

*sum of residual deviations, ** estimate of the number of parameters

Table S25. Network estimated effect sizes (95% confidence interval) for Vaccine efficacy (Bayesian random effects model)

The columns present the row drug class compared to the column vaccine class. The rows present the row vaccine class compared to the column vaccine class. The effect estimates are expressed as risk ratio and 95% confidence intervals. For example, Vaccine efficacy with AZD1222 compared to Gam-COVID-Vac is -0.84 (95% confidence interval -4.31 to 5.68).

AZD1222									
0.84 (-4.30, 5.68)	Gam-COVID-Vac								
-0.25 (-5.45, 5.46)	-1.15 (-6.42, 4.23)	AD26.COV.2							
1.54 (-3.62, 5.64)	0.61 (-3.97, 4.71)	1.71 (-3.56, 6.60)	mRNA-1273						
0.10 (-4.32, 5.33)	-0.89 (-4.37, 3.85)	0.34 (-3.93, 4.31)	-1.44 (-5.24, 3.10)	NVX-CoV2373					
1.41 (-3.13, 7.47)	0.47 (-3.83, 6.31)	1.77 (-3.30, 7.00)	0.04 (-4.72, 5.91)	1.43 (-2.57, 6.34)	BNT162b2				
-1.51 (-5.13, 2.69)	-2.44 (-5.55, 1.38)	-1.26 (-4.62, 2.45)	-2.90 (-6.06, 0.80)	-1.54 (-3.93, 0.86)	-3.03 (-6.72, 0.33)	Placebo			
-0.08 (-4.76, 6.03)	-0.90 (-5.32, 4.63)	0.00 (-4.33, 6.13)	-1.45 (-5.98, 3.68)	-0.06 (-4.86, 4.79)	-1.64 (-6.48, 4.23)	1.36 (-1.77, 5.38)	BBIBP-CorV		
-0.50 (-5.96, 4.58)	-1.46 (-6.22, 4.32)	-0.30 (-5.51, 4.87)	-1.99 (-6.52, 2.61)	-0.64 (-4.97, 4.23)	-2.04 (-7.26, 3.65)	0.95 (-2.88, 4.13)	-0.53 (-5.53, 3.82)	WIV04	
-0.93 (-5.28, 4.45)	-1.73 (-6.09, 3.49)	-0.66 (-6.07, 4.57)	-2.31 (-6.69, 3.70)	-0.95 (-4.62, 3.34)	-2.23 (-7.91, 3.37)	0.66 (-2.92, 4.38)	-0.76 (-6.19, 4.13)	-0.24 (-4.88, 4.85)	Corona Vac

Table S26. Network estimated effect sizes (95% confidence interval) for Neutralizing antibody responses to live SARS-CoV-2 (Bayesian random effects model)

The columns present the row drug class compared to the column vaccine class. The rows present the row vaccine class compared to the column vaccine class. The effect estimates are expressed as mean difference and 95% confidence intervals. For example, Neutralizing antibody responses to live SARS-CoV-2 with ZF2001 compared to Ad5-nCoV is -6.55 (95% confidence interval -48.64 to 34.23).

ZF2001											
-7.35 (-42.14, 32.00)	Ad5-nCoV										
-28.00 (-82.52, 20.34)	-25.37 (-79.36, 32.78)	Gam-COVID-Vac									
-205.94 (-267.09, -149.13)	-198.92 (-266.63, -137.49)	-178.78 (-262.34, -110.56)	AD26.COV.2								
-1590.70 (-1665.54, -1528.96)	-1584.26 (-1663.68, -1516.06)	-1560.07 (-1647.57, -1487.15)	-1382.88 (-1470.02, -1304.46)	mRNA-1273							
-1358.88 (-1700.05, -1012.33)	-1347.45 (-1691.32, -1003.73)	-1331.32 (-1673.13, -974.05)	-1142.71 (-1500.90, -816.13)	229.32 (-124.03, 589.64)	NVX-CoV2373						
13.04 (-7.63, 33.34)	19.42 (-11.02, 45.03)	41.86 (-9.43, 88.42)	220.00 (165.65, 276.33)	1602.02 (1546.81, 1671.78)	1368.92 (1021.49, 1718.73)	Placebo					
-6.45 (-34.95, 20.31)	-0.41 (-33.86, 31.87)	22.75 (-31.41, 70.54)	199.44 (141.13, 259.46)	1582.92 (1518.69, 1656.73)	1347.87 (1006.56, 1699.23)	-19.47 (-38.37, -2.01)	CoV2 preS dTM				
-138.94 (-189.42, -86.93)	-132.67 (-187.67, -81.44)	-108.95 (-181.26, -51.83)	67.42 (-5.24, 143.90)	1452.26 (1377.33, 1530.51)	1219.64 (856.48, 1564.58)	-152.03 (-196.56, -108.14)	-131.74 (-177.84, -86.16)	BBIBP-CorV			
-104.67 (-144.81, -71.68)	-98.18 (-147.03, -59.09)	-76.78 (-140.90, -22.20)	101.43 (33.76, 164.45)	1483.93 (1415.76, 1559.79)	1253.69 (905.85, 1603.05)	-117.45 (-154.88, -89.29)	-97.07 (-136.30, -63.56)	34.73 (-23.76, 81.78)	WIV04		
-21.35 (-50.11, 3.35)	-15.75 (-50.04, 16.68)	6.41 (-43.47, 55.09)	184.73 (128.51, 243.25)	1566.50 (1504.53, 1637.01)	1335.99 (985.77, 1677.18)	-35.21 (-49.96, -21.35)	-14.88 (-36.43, 8.57)	116.33 (74.31, 163.36)	82.66 (51.86, 121.24)	Corona Vac	
-56.79 (-89.41, -29.18)	-50.75 (-94.54, -18.83)	-31.22 (-77.74, 19.12)	147.05 (86.57, 210.67)	1531.66 (1470.54, 1603.40)	1301.58 (959.79, 1634.95)	-69.44 (-96.50, -52.78)	-49.93 (-82.18, -24.18)	80.97 (30.63, 126.30)	46.76 (8.34, 88.86)	-35.68 (-64.81, -13.52)	MINHAI

Table S27. Network estimated effect sizes (95% confidence interval) for Any local AR of first vaccination (Bayesian random effects model)

The columns present the row drug class compared to the column vaccine class. The rows present the row vaccine class compared to the column vaccine class. The effect estimates are expressed as odds ratio and 95% confidence intervals. For example, Any local AR of first vaccination with mRNA-1273 compared to NVX-CoV2373 is 1.94 (95% confidence interval 1.07 to 2.69).

mRNA-1273				
1.94 (1.07, 2.69)	NVX-CoV2373			
1.15 (0.24, 2.01)	-0.79 (-2.06, 0.30)	BNT162b2		
3.05 (2.53, 3.50)	1.12 (0.46, 1.86)	1.89 (1.07, 2.82)	Placebo	
2.20 (1.54, 3.02)	0.27 (-0.44, 1.25)	1.06 (0.23, 2.26)	-0.85 (-1.27, -0.25)	Corona Vac

Table S28. Network estimated effect sizes (95% confidence interval) for Any local AR of second vaccination (Bayesian random effects model)

The columns present the row drug class compared to the column vaccine class. The rows present the row vaccine class compared to the column vaccine class. The effect estimates are expressed as odds ratio and 95% confidence intervals. For example, Any local AR of second vaccination with mRNA-1273 compared to NVX-CoV2373 is 0.41 (95% confidence interval -1.10 to 2.00).

mRNA-1273				
0.41<br>(-1.10, 2.00)	NVX-CoV2373			
0.49<br>(-2.04, 2.75)	0.03<br>(-2.58, 2.26)	BNT162b2		
2.46<br>(1.66, 3.55)	2.08<br>(0.95, 3.09)	2.08<br>(-0.02, 4.17)	Placebo	
1.34<br>(-0.13, 2.66)	0.95<br>(-0.54, 2.45)	0.93<br>(-1.24, 3.56)	-1.14<br>(-2.26, -0.19)	Corona Vac

Table S29. Network estimated effect sizes (95% confidence interval) for Any systemic AR of first vaccination (Bayesian random effects model)

The columns present the row drug class compared to the column vaccine class. The rows present the row vaccine class compared to the column vaccine class. The effect estimates are expressed as odds ratio and 95% confidence intervals. For example, Any systemic AR of first vaccination with mRNA-1273 compared to NVX-CoV2373 is 0.13 (95% confidence interval -0.17 to 0.44).

mRNA-1273				
0.13<br>(-0.17, 0.44)	NVX-CoV2373			
0.12<br>(-0.30, 0.56)	-0.01<br>(-0.42, 0.42)	BNT162b2		
0.50<br>(0.31, 0.73)	0.37<br>(0.14, 0.60)	0.39<br>(0.07, 0.77)	Placebo	
0.14<br>(-0.19, 0.46)	0.01<br>(-0.33, 0.34)	0.01<br>(-0.37, 0.43)	-0.37<br>(-0.63, -0.13)	Corona Vac

Table S30. Network estimated effect sizes (95% confidence interval) for Any systemic AR of second vaccination (Bayesian random effects model)

The columns present the row drug class compared to the column vaccine class. The rows present the row vaccine class compared to the column vaccine class. The effect estimates are expressed as odds ratio and 95% confidence intervals. For example, Any systemic AR of second vaccination with mRNA-1273 compared to NVX-CoV2373 is -0.30 (95% confidence interval -1.42 to 0.69).

mRNA-1273				
-0.30<br>(-1.42, 0.69)	NVX-CoV2373			
-0.58<br>(-2.06, 1.00)	-0.30<br>(-1.84, 1.49)	BNT162b2		
0.63<br>(-0.11, 1.33)	0.96<br>(0.14, 1.69)	1.23<br>(-0.43, 2.56)	Placebo	
0.59<br>(-0.78, 1.56)	0.88<br>(-0.35, 1.98)	1.18<br>(-0.81, 2.53)	-0.07<br>(-0.96, 0.67)	Corona Vac

Table S31. Network estimated effect sizes (95% confidence interval) for total dose of Any local AR (Bayesian random effects model)

The columns present the row drug class compared to the column vaccine class. The rows present the row vaccine class compared to the column vaccine class. The effect estimates are expressed as odds ratio and 95% confidence intervals. For example, Any local AR of total dose with AZD1222 compared to ZF2001 is -1.47 (95% confidence interval -3.01 to 0.12).

AZD1222								
-1.47<br>(-3.01, 0.12)	ZF2001							
-2.99<br>(-4.61, -0.98)	-1.48<br>(-2.91, -0.12)	Ad5-nCoV						
-3.45<br>(-5.53, -1.76)	-1.94<br>(-3.63, -0.27)	-0.45<br>(-2.30, 1.22)	mRNA-1273					
0.02<br>(-1.39, 1.47)	1.50<br>(0.73, 2.33)	3.02<br>(1.92, 4.12)	3.47<br>(2.12, 4.99)	Placebo				
-0.79<br>(-2.35, 0.67)	0.70<br>(-0.56, 1.75)	2.17<br>(0.49, 3.58)	2.63<br>(1.00, 4.39)	-0.79<br>(-1.80, -0.02)	BBIBP-CorV			
0.39<br>(-1.39, 2.06)	1.87<br>(0.54, 2.98)	3.39<br>(1.79, 4.72)	3.82<br>(2.13, 5.51)	0.37<br>(-0.72, 1.24)	1.16<br>(-0.18, 2.56)	WIV04		
-0.92<br>(-2.40, 0.48)	0.57<br>(-0.31, 1.59)	2.08<br>(0.75, 3.43)	2.56<br>(1.12, 4.06)	-0.93<br>(-1.58, -0.34)	-0.14<br>(-1.11, 1.11)	-1.28<br>(-2.36, -0.00)	Corona Vac	
-0.23<br>(-1.98, 1.42)	1.27<br>(-0.01, 2.59)	2.71<br>(1.30, 4.19)	3.22<br>(1.55, 5.01)	-0.24<br>(-1.12, 0.60)	0.53<br>(-0.55, 1.90)	-0.62<br>(-1.90, 0.76)	0.71<br>(-0.45, 1.76)	MINHAI

Table S32. Network estimated effect sizes (95% confidence interval) for total dose of Any systemic AR (Bayesian random effects model)

The columns present the row drug class compared to the column vaccine class. The rows present the row vaccine class compared to the column vaccine class. The effect estimates are expressed as odds ratio and 95% confidence intervals. For example, Any systemic AR of total dose with ZF2001 compared to mRNA-1273 is -1.63 (95% confidence interval -2.63 to 0.66).

ZF2001						
-1.63<br>(-2.63, -0.66)	mRNA-1273					
0.23<br>(-0.37, 0.87)	1.91<br>(1.02, 2.59)	Placebo				
0.50<br>(-0.20, 1.41)	2.15<br>(1.25, 3.08)	0.27<br>(-0.23, 0.85)	BBIBP-CorV			
0.32<br>(-0.54, 1.33)	1.98<br>(0.78, 2.99)	0.10<br>(-0.54, 0.70)	-0.18<br>(-1.21, 0.50)	WIV04		
0.39<br>(-0.27, 1.17)	2.07<br>(1.10, 2.93)	0.17<br>(-0.31, 0.70)	-0.10<br>(-0.85, 0.57)	0.09<br>(-0.69, 0.88)	Corona Vac	
-0.14<br>(-1.23, 0.87)	1.49<br>(0.30, 2.50)	-0.39<br>(-1.25, 0.34)	-0.68<br>(-1.89, 0.22)	-0.51<br>(-1.49, 0.49)	-0.56<br>(-1.55, 0.31)	MINHAI

Table S33. Network estimated effect sizes (95% confidence interval) for Unsolicited AR (Bayesian random effects model)

The columns present the row drug class compared to the column vaccine class. The rows present the row vaccine class compared to the column vaccine class. The effect estimates are expressed as odds ratio and 95% confidence intervals. For example, unsolicited AR with ZF2001 compared to Ad5-nCoV is -0.26 (95% confidence interval -1.13 to 0.52).

ZF2001										
-0.26<br>(-1.13, 0.52)	Ad5-nCoV									
-0.31<br>(-0.95, 0.28)	-0.01<br>(-0.61, 0.73)	AD26.COV.S								
-0.85<br>(-1.54, 0.30)	-0.56<br>(-1.21, 0.14)	-0.55<br>(-0.98, 0.17)	mRNA-1273							
-1.20<br>(-1.92, -0.58)	-0.97<br>(-1.63, -0.26)	-0.94<br>(-1.36, -0.50)	-0.41<br>(-0.81, 0.07)	NVX-<br>CoV2373						
-0.18<br>(-0.81, 0.31)	0.10<br>(-0.46, 0.73)	0.11<br>(-0.24, 0.40)	0.65<br>(0.44, 0.89)	1.05<br>(0.72, 1.37)	Placebo					
-0.91<br>(-1.64, -0.25)	-0.56<br>(-1.47, 0.19)	-0.58<br>(-1.23, -0.01)	-0.03<br>(-0.60, 0.55)	0.38<br>(-0.30, 0.96)	-0.68<br>(-1.27, -0.19)	CoV preS<br>dTM				
-0.16<br>(-0.82, 0.33)	0.11<br>(-0.50, 0.82)	0.11<br>(-0.26, 0.58)	0.65<br>(0.38, 1.06)	1.06<br>(0.67, 1.48)	-0.01<br>(-0.19, 0.32)	0.68<br>(0.20, 1.29)	BBIBP-CorV			
-0.25<br>(-0.91, 0.31)	0.05<br>(-0.61, 0.73)	0.06<br>(-0.46, 0.51)	0.60<br>(0.22,. 0.99)	1.00<br>(0.55, 1.43)	-0.05<br>(-0.35, 0.22)	0.62<br>(0.06, 1.25)	-0.05<br>(-0.55, 0.25)	WIV04		
0.10<br>(-0.99, 0.83)	0.35<br>(-0.761, 1.23)	0.34<br>(-0.71, 1.08)	0.93<br>(-0.18, 1.64)	1.31<br>(0.20, 2.03)	0.36<br>(-0.74, 0.96)	0.98<br>(-0.17, 1.89)	0.27<br>(-0.84, 0.97)	0.32<br>(-0.78, 1.06)	Corona Vac	
0.78<br>(-0.74, 2.24)	0.93<br>(-0.36, 2.80)	0.93<br>(-0.46, 2.79)	1.48<br>(0.07, 3.33)	1.82<br>(0.43, 3.78)	0.79<br>(-0.56, 2.64)	1.54<br>(0.13, 3.27)	0.81<br>(-0.58, 2.67)	0.85<br>(-0.47, 2.72)	0.88<br>(-1.11, 2.24)	MINHAI

Table S34. Network estimated effect sizes (95% confidence interval) for SAE (Bayesian random effects model)

The columns present the row drug class compared to the column vaccine class. The rows present the row vaccine class compared to the column vaccine class. The effect estimates are expressed as odds ratio and 95% confidence intervals. For example, SAE with AZD1222 compared to ZF2001 is -0.21 (95% confidence interval -1.53 to 1.07).

AZD1222												
-0.21 (-1.53, 1.07)	ZF2001											
0.46 (-2,00 3.21)	0.71 (-1.24, 2.89)	Ad5-nCoV										
0.56 (-0.39, 4.05)	0.73 (-0.46, 1.24)	0.11 (-1.90, 2.00)	Gam-COVID-Vac									
0.26 (-0.76, 2.63)	0.39 (-0.70, 1.46)	-0.18 (-2.17, 1.70)	-0.43 (-0.90, 0.56)	AD26.COV.S								
0.33 (-0.58, 1.21)	0.48 (-0.51, 1.41)	-0.10 (-2.13, 1.90)	-0.23 (-0.24, 0.12)	0.12 (-0.46, 0.97)	mRNA-1273							
0.14 (-0.82, 0.99)	0.23 (-0.81, 1.29)	-0.30 (-2.39, 1.60)	-0.46 (-1.76, 0.11)	-0.16 (-0.76, 0.54)	-0.19 (-0.80, 0.41)	NVX-CoV2373						
-0.03 (-0.84, 0.65)	0.15 (-0.87, 2.09)	-0.49 (-2.47, 1.49)	-0.59 (-1.14, -0.03)	-0.29 (-0.64, 0.12)	-0.40 (-0.80, 0.06)	-0.20 (-0.69, 0.26)	BNT162b2					
0.09 (-0.70, 0.76)	0.24 (-0.76, 1.26)	-0.30 (-2.59, 1.87)	-0.50 (-0.96, 0.05)	-0.16 (-0.47, 0.19)	-0.26 (-0.60, 0.10)	-0.04 (-0.46, 0.43)	0.18 (-0.24, 0.39)	Placebo				
0.16 (-1.12, 1.39)	0.34 (-1.05, 1.46)	-0.21 (-2.69, 1.46)	-0.42 (-1.45, 0.71)	-0.10 (-1.64, 0.76)	-0.16 (-1.20, 0.85)	0.06 (-1.03, 1.13)	0.21 (-0.79, 1.11)	0.08 (-0.93, 1.06)	CoV2 preS dTM			
0.43 (-0.40, 1.11)	0.53 (-0.41, 1.50)	-0.02 (-2.07, 1.49)	-0.14 (-0.73, 0.46)	0.20 (-0.21, 0.86)	0.10 (-0.40, 0.60)	0.33 (-0.29, 0.74)	0.43 (0.01, 0.84)	0.34 (0.12, 0.87)	0.24 (-0.70, 1.31)	BBIBP-CorV		
0.33 (-0.56, 1.15)	0.41 (-0.55, 1.49)	-0.25 (-2.98, 3.26)	-0.23 (-0.86, 0.39)	0.09 (-0.40, 0.35)	-0.03 (-0.49, 0.44)	0.23 (-0.46, 0.89)	0.37 (-0.04, 0.79)	0.24 (-0.64, 0.11)	0.24 (-0.90, 1.35)	-0.13 (-0.79, 0.43)	WIV04	
0.07 (-0.89, 0.99)	0.20 (-0.90, 1.37)	-0.37 (-2.56, 1.88)	-0.50 (-1.42, 0.86)	-0.15 (-0.71, 0.40)	-0.24 (-0.88, 0.26)	-0.07 (-0.98, 0.46)	0.13 (-0.38, 0.72)	-0.04 (-0.56, 0.50)	-0.10 (-1.20, 0.49)	-0.39 (-0.67, 0.18)	-0.22 (-0.43, 0.46)	Corona Vac

Table S35. Certainty of evidence evaluated with GRADE framework of safety

Comparisons(vs. Placebo)	Study No.	Effect size (95% CI)	Study design	GRADE
Any local adverse reaction of total vaccination, Relative risk				
AZD1222	1	1.04 (0.33, 3.23)	RCT	⊕⊕⊕○ Moderate
mRNA-1273	1	33.57 (10.90, 103.43)	RCT	⊕⊕○○ Low
BBIBP-CorV	2	1.86 (0.84, 4.09)	RCT	⊕⊕⊕○ Moderate
WIV04	2	0.72 (0.31, 1.70)	RCT	⊕⊕⊕○ Moderate
CoronaVac	1	2.44 (1.41, 4.23)	RCT	⊕⊕⊕○ Moderate
ZF2001	1	4.23 (2.08, 8.59)	RCT	⊕⊕⊕○ Moderate
Ad5-nCoV	1	19.52 (7.77, 49.00)	RCT	⊕⊕○○ Low
MINHAI	1	1.23 (0.56, 2.68)	RCT	⊕⊕⊕○ Moderate
Any systemic adverse reaction of total vaccination				
ZF2001	1	1.27 (0.77, 2.08)	RCT	⊕⊕⊕○ Moderate
mRNA-1273	1	6.69 (3.82, 11.71)	RCT	⊕⊕⊕○ Moderate
BBIBP-CorV	1	0.75 (0.48, 1.17)	RCT	⊕⊕⊕○ Moderate
WIV04	1	0.92 (0.55, 1.54)	RCT	⊕⊕⊕○ Moderate
Corona vac	1	0.99 (0.64, 1.53)	RCT	⊕⊕⊕○ Moderate
MINHAI	1	1.37 (0.64, 2.90)	RCT	⊕⊕⊕○ Moderate
Any local adverse reaction of first vaccination, Relative risk				
NVX-CoV2373	1	4.19 (2.51, 6.98)	RCT	⊕⊕○○ Low
BNT162b1	1	6.86 (3.20, 14.69)	RCT	⊕⊕⊕○ Moderate
mRNA-1273	1	20.25 (13.67, 30.01)	RCT	⊕⊕⊕○ Moderate
CoronaVac	1	2.20 (1.50, 3.22)	RCT	⊕⊕⊕○ Moderate
Any local adverse reaction of second vaccination, Relative risk				
NVX-CoV2373	1	7.34 (2.46, 21.93)	RCT	⊕⊕○○ Low
BNT162b1	1	7.44 (1.14, 48.50)	RCT	⊕⊕○○ Low
mRNA-1273	1	11.03 (4.61, 26.41)	RCT	⊕⊕⊕○ Moderate
CoronaVac	1	2.80 (1.15, 6.85)	RCT	⊕⊕⊕○ Moderate
Any systemic adverse reaction of first vaccination, Relative risk				
NVX-CoV2373	3	1.44 (1.25, 1.66)	RCT	⊕⊕⊕○ Moderate
BNT162b1	1	1.46 (1.20, 1.78)	RCT	⊕⊕⊕○ Moderate
mRNA-1273	2	1.66 (1.59, 1.74)	RCT	⊕⊕⊕⊕ High
CoronaVac	2	2.20 (1.50, 3.22)	RCT	⊕⊕⊕⊕ High
Any systemic adverse reaction of second vaccination, Relative risk				
NVX-CoV2373	3	2.58 (1.04, 6.43)	RCT	⊕⊕○○ Low
BNT162b1	1	3.44 (0.55, 21.45)	RCT	⊕⊕○○ Low
mRNA-1273	2	1.88 (0.81, 4.35)	RCT	⊕⊕⊕○ Moderate
CoronaVac	2	0.99 (0.39, 2.52)	RCT	⊕⊕⊕⊕ High

Table S35. Certainty of evidence evaluated with GRADE framework of safety (continue)

Comparisons(vs. Placebo)	Study No.	Effect size (95% CI)	Study design	GRADE
Unsolicited adverse reaction, Relative risk				
ZF2001	1	0.74 (0.43, 1.28)	RCT	⊕⊕○○ Low
Ad5-nCoV	1	1.14 (0.59, 2.18)	RCT	⊕⊕⊕○ Moderate
Ad26.COV2.S	1	1.10 (0.95, 1.27)	RCT	⊕⊕⊕○ Moderate
mRNA-1273	2	1.89 (1.72, 2.07)	RCT	⊕⊕⊕⊕ High
NVX-CoV2373	1	2.79 (2.08, 3.76)	RCT	⊕⊕○○ Low
CoV2 preS dTM	1	1.93 (1.19, 3.14)	RCT	⊕⊕⊕○ Moderate
BBIBP-CorV	2	1.01 (0.94, 1.08)	RCT	⊕⊕⊕⊕ High
WIV04	1	1.05 (0.98, 1.12)	RCT	⊕⊕⊕○ Moderate
Corona vac	1	0.79 (0.26, 2.37)	RCT	⊕⊕○○ Low
MINHAI	1	0.49 (0.12, 2.00)	RCT	⊕⊕○○ Low
Serious adverse event, Relative risk				
AZD1222	1	1.08 (0.50, 2.30)	RCT	⊕⊕○○ Low
ZF2001	1	1.25 (0.48, 3.26)	RCT	⊕⊕○○ Low
Ad5-nCoV	1	0.70 (0.10, 4.97)	RCT	⊕⊕○○ Low
Gam-COVID-Vac	1	0.63 (0.39, 1.04)	RCT	⊕⊕⊕○ Moderate
Ad26.COV2.S	1	0.87 (0.65, 1.16)	RCT	⊕⊕⊕○ Moderate
mRNA-1273	1	0.79 (0.58, 1.08)	RCT	⊕⊕⊕○ Moderate
NVX-CoV2373	1	0.98 (0.64, 1.48)	RCT	⊕⊕○○ Low
BNT162b1	1	1.14 (0.88, 1.46)	RCT	⊕⊕⊕○ Moderate
CoV2 preS dTM	1	0.94 (0.36, 2.47)	RCT	⊕⊕○○ Low
BBIBP-CorV	2	0.72 (0.52, 1.00)	RCT	⊕⊕⊕⊕ High
WIV04	2	0.80 (0.58, 1.11)	RCT	⊕⊕⊕⊕ High
Corona vac	2	1.03 (0.63, 1.67)	RCT	⊕⊕⊕⊕ High

High quality: We are extremely certain that the actual effect closely matches the effect estimate.

Moderate quality: We have a mediocre level of confidence in the impact estimate; the genuine effect is likely to be similar to the estimate, but there is a chance that it will be significantly different.

Low quality: We have little faith in the impact estimate. The actual effect could differ significantly from the estimated effect.

Reference

1. Dias S, Welton NJ, Sutton AJ, Caldwell DM, Lu G, Ades AE. Evidence synthesis for decision making 4: inconsistency in networks of evidence based on randomized controlled trials. *Med Decis Making* 2013; **33**(5): 641-56.
2. Dias S, Sutton AJ, Ades AE, Welton NJ. Evidence synthesis for decision making 2: a generalized linear modeling framework for pairwise and network meta-analysis of randomized controlled trials. *Med Decis Making* 2013; **33**(5): 607-17.
3. Verardi S, Casciani CU, Nicora E, et al. A multicentre study on LMW-heparin effectiveness in preventing postsurgical thrombosis. *Int Angiol* 1988; **7**(3 Suppl): 19-24.
4. Lumley T. Network meta-analysis for indirect treatment comparisons. *Stat Med* 2002; **21**(16): 2313-24.
5. Jansen JP, Fleurence R, Devine B, et al. Interpreting indirect treatment comparisons and network meta-analysis for health-care decision making: report of the ISPRR Task Force on Indirect Treatment Comparisons Good Research Practices: part 1. *Value Health* 2011; **14**(4): 417-28.
6. Hoaglin DC, Hawkins N, Jansen JP, et al. Conducting indirect-treatment-comparison and network-meta-analysis studies: report of the ISPRR Task Force on Indirect Treatment Comparisons Good Research Practices: part 2. *Value Health* 2011; **14**(4): 429-37.
7. Bucher HC, Guyatt GH, Griffith LE, Walter SD. The results of direct and indirect treatment comparisons in meta-analysis of randomized controlled trials. *J Clin Epidemiol* 1997; **50**(6): 683-91.
8. Dias S, Sutton AJ, Welton NJ, Ades AE. Evidence synthesis for decision making 3: heterogeneity--subgroups, meta-regression, bias, and bias-adjustment. *Med Decis Making* 2013; **33**(5): 618-40.
9. Schwarzer G, Carpenter JR, Rücker G. Network Meta-Analysis. Meta-Analysis with R. Cham: Springer International Publishing; 2015: 187-216.
10. Dobler CC, Wilson ME, Murad MH. A pulmonologist's guide to understanding network meta-analysis. *Eur Respir J* 2018; **52**(1).
